# Avian influenza viruses in New Zealand wild birds, with an emphasis on subtypes H5 and H7: Their distinctive epidemiology and genomic properties

**DOI:** 10.1371/journal.pone.0303756

**Published:** 2024-06-03

**Authors:** Wlodek L. Stanislawek, Toni Tana, Thomas G. Rawdon, Susan C. Cork, Kylie Chen, Hammed Fatoyinbo, Naomi Cogger, Richard J. Webby, Robert G. Webster, Maree Joyce, Mary Ann Tuboltsev, Della Orr, Sylvia Ohneiser, Jonathan Watts, Adrian C. Riegen, Matthew McDougall, David Klee, Joseph S. O’Keefe

**Affiliations:** 1 Ministry for Primary Industries, Upper Hutt, New Zealand; 2 Department of Ecosystem & Public Health, Faculty of Veterinary Medicine, University of Calgary, Calgary, Alberta, Canada; 3 Department of Computational Biology, University of Auckland, Auckland, New Zealand; 4 EpiCentre, School of Veterinary Science, Massey University, Palmerston North, New Zealand; 5 Department of Infectious Diseases, St. Jude Children’s Research Hospital, Memphis, Tennessee, United States of America; 6 Manaaki Whenua—Landcare Research, Lincoln, New Zealand; 7 Pūkorokoro Miranda Naturalists’ Trust, Mirnda, New Zealand; 8 Fish and Game, Rotorua, New Zealand; Foshan University School of Life Science and Engineering, CHINA

## Abstract

The rapid spread of highly pathogenic avian influenza (HPAI) A (H5N1) viruses in Southeast Asia in 2004 prompted the New Zealand Ministry for Primary Industries to expand its avian influenza surveillance in wild birds. A total of 18,693 birds were sampled between 2004 and 2020, including migratory shorebirds (in 2004–2009), other coastal species (in 2009–2010), and resident waterfowl (in 2004–2020). No avian influenza viruses (AIVs) were isolated from cloacal or oropharyngeal samples from migratory shorebirds or resident coastal species. Two samples from red knots *(Calidris canutus)* tested positive by influenza A RT-qPCR, but virus could not be isolated and no further characterization could be undertaken. In contrast, 6179 samples from 15,740 mallards (*Anas platyrhynchos*) tested positive by influenza A RT-qPCR. Of these, 344 were positive for H5 and 51 for H7. All H5 and H7 viruses detected were of low pathogenicity confirmed by a lack of multiple basic amino acids at the hemagglutinin (HA) cleavage site. Twenty H5 viruses (six different neuraminidase [NA] subtypes) and 10 H7 viruses (two different NA subtypes) were propagated and characterized genetically. From H5- or H7-negative samples that tested positive by influenza A RT-qPCR, 326 AIVs were isolated, representing 41 HA/NA combinations. The most frequently isolated subtypes were H4N6, H3N8, H3N2, and H10N3. Multivariable logistic regression analysis of the relations between the location and year of sampling, and presence of AIV in individual waterfowl showed that the AIV risk at a given location varied from year to year. The H5 and H7 isolates both formed monophyletic HA groups. The H5 viruses were most closely related to North American lineages, whereas the H7 viruses formed a sister cluster relationship with wild bird viruses of the Eurasian and Australian lineages. Bayesian analysis indicates that the H5 and H7 viruses have circulated in resident mallards in New Zealand for some time. Correspondingly, we found limited evidence of influenza viruses in the major migratory bird populations visiting New Zealand. Findings suggest a low probability of introduction of HPAI viruses via long-distance bird migration and a unique epidemiology of AIV in New Zealand.

## Introduction

The dissemination of highly pathogenic avian influenza (HPAI) H5N1 viruses throughout Southeast Asia in 2004 prompted the New Zealand Ministry for Primary Industries (MPI), previously known as the Ministry of Agriculture and Forestry, to expand its avian influenza (AI) surveillance in migratory shorebirds (waders), coastal species, and resident waterfowl to examine the role of these populations as potential hosts of HPAI H5 and H7 viruses. To date, no HPAI viruses have been isolated from New Zealand poultry or wild birds, although there are potential pathways for virus entry through the illegal importation of live birds or bird products or via migratory wild birds.

New Zealand lies at the southeastern extremity of the East Asian–Australasian migratory bird flyway. However, given New Zealand’s geographical isolation, relatively few migratory shorebirds reach the country from the northern hemisphere, and only three such species occur in significant numbers: the bar-tailed godwit (*Limosa lapponica*), the red (lesser) knot (*Calidris canutus*), and the ruddy turnstone (*Arenaria interpres*). These species make up 90% of the approximately 200,000 migratory shorebirds that visit New Zealand each year [1–3].

**Fig 1:**
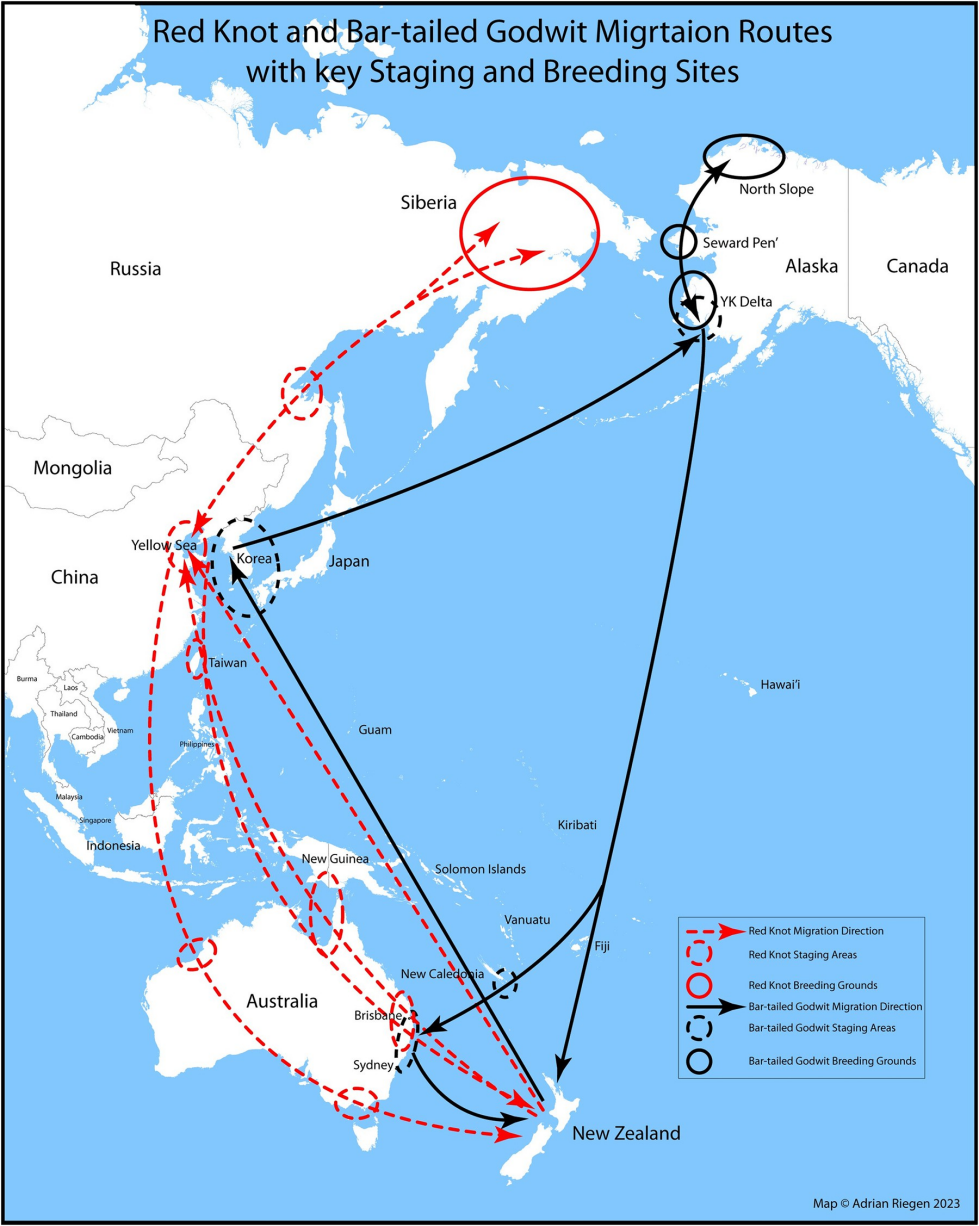
Map of migration routes of waders to and from New Zealand (Credit: Adrian Riegan, Pūkorokoro Miranda Naturalists’ Trust).

Although migratory routes continue to be defined, most bar-tailed godwits fly directly to New Zealand from Alaska or via eastern Australia [4, 5]. Red knots have stopovers in East Asia, with some also stopping in Australia [3, 6], and ruddy turnstones have stops in the Pacific and Australia before reaching New Zealand [7] (Fig 1). On arrival, migratory birds infected with avian influenza viruses (AIVs) could shed virus and contaminate the environment they share with resident shorebirds, gulls, and waterfowl species.

Wild aquatic birds of the orders *Anseriformes* (ducks, geese, and swans) and, to a lesser extent, *Charadriiformes* (gulls, terns, and waders) are the major natural reservoir for AIVs [8–10]. There is evidence that wild waterfowl movements can facilitate local [11] and long-distance spread of AIVs, including HPAI viruses [12, 13]. However, waterfowl do not migrate to or from New Zealand, except for intermittent single or small numbers of vagrants from Australia and the Pacific Islands [1]. Therefore, waterfowl are an unlikely host for introducing AIV to New Zealand, but resident populations could become reservoirs for any newly introduced AIVs.

To guide the implementation of enhanced wild bird surveillance for novel, new, and emerging strains of AIV in New Zealand, an interdisciplinary team including virologists, epidemiologists, and ornithologists was assembled. Over 17 years, the study carried out annual surveys, employing both virological and serological techniques, to assess the presence and prevalence of avian influenza in migratory shorebirds and resident waterfowl. The primary objective of the study was to understand the strains of AIV circulating in New Zealand and to evaluate the risk of novel AIVs (particularly H5 and H7 subtypes) being introduced through the East Asia–Australasian flyway. In addition, the study aimed to develop an understanding of the risk posed to New Zealand’s commercial poultry industry by endemic H5- and H7-subtype viruses circulating in resident reservoir species, particularly waterfowl. The study focused particularly on H5 and H7 subtypes, given the recognition by the World Organisation for Animal Health (WOAH) of their relevance to animal and human health. The emerging global impact of the clade 2.3.4.4b A (H5N1) viruses highlights the relevance of this work to New Zealand’s national biosecurity systems and preparedness planning.

## Materials and methods

### Study design

A risk-based approach was implemented, focused on the arrival sites of migratory birds. Migratory species were sampled soon after their arrival in the spring (September–November), whereas resident waterfowl were sampled in mid-to-late summer (January–February), with a particular focus on coastal areas near migratory shorebird arrival sites.

Other avian species were sampled on an opportunistic basis (see S1 Table).

The mid-summer sampling period for waterfowl allowed time for cross-transfer of AIVs from arriving migratory birds to local waterfowl. The study was designed to balance the numbers that it was practical to collect at a single location over 1 or 2 days [S1 Table] while targeting risk both temporally and spatially. It was planned to collect oropharyngeal and cloacal swabs from approximately 300 waterfowl per site [S1 Table] to enable the detection of at least one sample that was positive for AIV/RNA at a 1% design prevalence (95% confidence) [14]. For 4 years (2004–2007), serological surveillance using a hemagglutination inhibition (HI) test was also implemented as supplementary testing alongside the AIV molecular determinations. Thirty blood samples from mallards were collected at each location [S1 Table] to enable the detection of at least one positive sample at a design prevalence of 10% (95% confidence) [14].

The minimum number of birds required to detect at least one positive sample with 95% confidence at the design prevalences detailed above was calculated for a large population (10,000 birds), using a probability formula adjusted for imperfect laboratory tests [14]. A sensitivity and specificity of 98.78% and 100%, respectively, for the molecular assay was used [15, 16], with 100% specificity reflecting the full process of testing with follow-up confirmatory testing by sequence analysis (described later). The specificity of the HI testing reflects the use of two antigens with the test results interpreted in series. The sensitivity and specificity of the HI test considered were 98.8% and 99.5%, respectively [17].

### Sample collection

Between 2004 and 2009, oropharyngeal and cloacal swabs were collected from migratory shorebirds soon after their arrival at selected sites in New Zealand. Birds were trapped using cannon nets and mist nets by the New Zealand Wader Study Group and Ornithological Society of New Zealand personnel and were released after banding and sampling.

**Fig 2:**
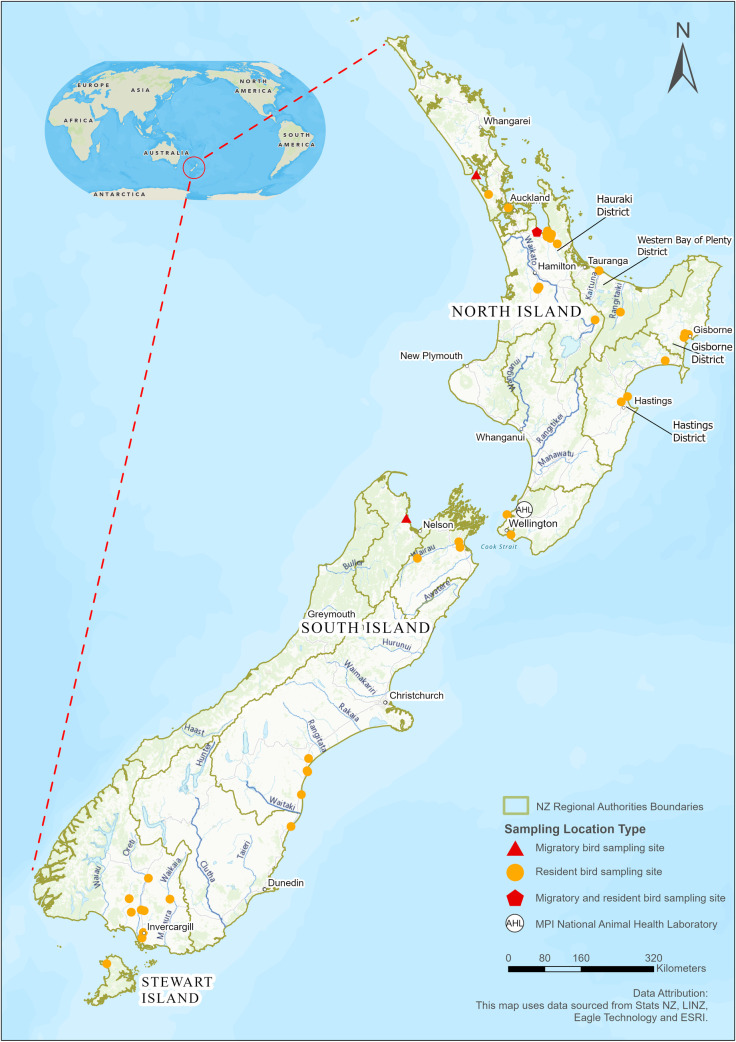
New Zealand sites where wild birds were captured for sample collection for avian influenza surveillance testing, 2004–2020.

All resident waterfowl were caught by Regional Fish and Game Council personnel, using walk-in funnel traps in coastal, wetland, and agricultural areas in the summer (January–February) (Fig 2; Table 1 and S1 Table). Samples from other species (e.g., penguins, shearwaters, and gulls) were collected with the assistance of the Department of Conservation (DOC) and wildlife management organizations. Most of the sampling was associated with other programs run by Fish and Game Councils, the DOC, or the Ornithological Society of New Zealand, which involved banding birds for ecological or wildlife management activities. The exception to this was gull trapping, which was contracted by wildlife management organizations.

**Table 1:** Results for avian influenza virus (AIV) in resident and migratory wild birds in New Zealand sampled between 2004–2020.

Year of sampling	Species	No. of birds sampled and type of sample		Influenza A RT-qPCR (positive//tested)	No. of RT-qPCR positives		AIV isolation results	
		C*	OP**		H5	H7	H5	H7
2004	Mallard (RW)	381	0	43/128	0	0	0	0
	Knot (MS)	222	0	0/74	-	-	-	-
	Godwit (MS)	66	0	0/22	-	-	-	-
2005	Mallard	1057	0	55/354	0	1	0	1
	Paradise (RW)	53	0	0/18	-	-	-	-
	Knot	219	0	0/73	-	-	-	-
	Godwit	65	0	0/22	-	-	-	-
2006	Mallard	568	0	63/190	0	0	0	0
	Knot	220	0	2/76	0	0	0	0
	Godwit	24	0	0/8	-	-	-	-
	Turnstone (MS)	14	0	0/5	-	-	-	-
2007	Mallard	1025	72	114/367	2	0	2	0
	Paradise	43	23	0/23	-	-	-	-
	Grey teal (RW)	50	31	12/28	0	0	0	0
	Knot	19	19	0/38	-	-	-	-
	Godwit	29	29	0/58	-	-	-	-
2008	Mallard	1047	0	323/349	37	0	8	0
	Paradise	50	0	0/16	-	-	-	-
	Knot	226	226	0/226	-	-	-	-
	Godwit	17	17	0/17	-	-	-	-
2009	Mallard	1035	1035	306/690	4	0	0	0
	Knot	69	69	0/46	-	-	-	-
	Godwit	79	79	0/54	-	-	-	-
	Black-billed gull (RW)	200	200	0/134	-	-	-	-
	Black-backed gull (RW)	30	30	0/20	-	-	-	-
2010	Mallard	795	795	337/532	5	17	0	2
	Wrybill (RW)	80	80	0/60	-	-	-	-
	Black-backed gull	288	288	0/192	-	-	-	-
	Black-billed gull	635	635	0/424	-	-	-	-
	Little blue penguin (RW)	109	109	0/72	-	-	-	-
	Yellow-eyed penguin (RW)	54	54	0/108	-	-	-	-
	Sooty shearwater (RW)	80	80	0/160	-	-	-	-
2011	Mallard	791	791	368/528	25	0	4	0
	Penguin	12		0/12				
2012	Mallard	764	764	429/764	9	0	0	0
2013	Mallard	960	960	459/960	46	0	1	0
2014	Mallard	880	880	416/880	7	0	0	0
2015	Mallard	1065	1065	314/1065	0	8	0	5
2016	Mallard	963	963	735/963	166	0	4	0
2017	Mallard	1175	1175	895/1175	0	3	0	1
2018	Mallard	950	950	590/950	4	21	0	1
2019	Mallard	1023	1023	333/1023	38	0	0	0
2020	Mallard	1261	1261	354/1261	1	1	1	0
**Total**	** **	**18693**		**6155/14155**	**344**	**51**	**20**	**10**

*C = cloacal swabs

**OP = oropharyngeal swabs; RW = resident waterfowl; MS = migratory shorebirds

Latin names of the birds tested: mallard ducks (*Anas platyrhynchos*), bar-tailed godwit (*Limosa lapponica*), red (lesser) knot (*Calidris canutus*), ruddy turnstone (*Arenaria interpres*), grey teal (*Anas gracilis*), paradise shelduck (*Ta)dorna variegate*), black-billed gull (*Chroicocephalus bulleri*), black-backed gull (*Larus Dominicans*), wrybill (*Ngutu pare*), lLittle blue penguin (*Eudyptula minor/kororā*), yellow-eyed penguin (*Megadyptes antipodes*), sooty shearwater (*Ardenna grisea*).

Oropharyngeal and cloacal swabs for virus isolation and RT-PCR analysis were collected by inserting one cotton swab into the trachea of a bird and a second swab into its cloaca and rotating the swabs to obtain visible contamination. Swabs were placed in 1.2 mL of transport medium containing 10,000 units/mL penicillin, 10 mg/mL streptomycin, 250 μg/mL gentamycin, 5000 units/mL myostatin, and 1% bovine serum albumin in isotonic phosphate-buffered saline (PBS) (pH 7.4). Samples were stored from 24 to 48 h at 4°C then transferred to a −80°C freezer until tested.

Oropharyngeal and cloacal swabs were collected from waterfowl, predominantly mallards, between 2004 and 2020 [Table 1 and S1 Table]. In addition, between 2004 and 2007, whole-blood samples (1–2 mL) were collected from waterfowl. Blood was collected from the wing or jugular vein, using a 21-gauge needle. Sera were allowed to clot at 37°C for 1–2 h or overnight at room temperature. The serum was separated from blood clots by centrifugation at 2000 × *g* for 10 min and stored at −20°C until tested. No blood was collected from waders or from other species because of welfare concerns.

The field access/permission to the sampling sites was given by the relevant organisations such as Fish & Game New Zealand, Department of Conservation New Zealand, Pūkorokoro Miranda Naturalists’ Trust New Zealand. All organisations conducted their own programs and provided us with the opportunity to collect samples alongside their work.

AgResearch Animal Ethics Committee, New Zealand provided permits for the full length of this study to collect samples from birds and carry out virus isolation in embryonated Disease Free eggs.

### Molecular testing

#### Screening samples using influenza A matrix gene real-time TaqMan RT-qPCR

RNA was extracted from oropharyngeal and cloacal swabs after centrifugation at 1000 × *g* for 10 min by using a QIAamp Viral RNA Mini Kit (QIAGEN) or a high-throughput X-tragene or NucleoMag kit (Macherey-Nagel) and a KingFisher automated purification system (Thermo-Fisher), used in accordance with the respective manufacturers’ instructions. Influenza A matrix gene real-time TaqMan RT-qPCR was performed using primers and a probe (Sigma) developed by Spackman et al. [18] and modified by Heine et al. [16].

Samples positive for AIV by influenza A matrix gene real-time TaqMan RT-qPCR were further tested using H5-specific and H7-specific TaqMan RT-qPCR [19, 20]. All RT-qPCR analyses were performed in a 25-μL volume (20 μL of RT-qPCR mix and 5 μL of nucleic acid) using a SuperScript III One-Step RT-qPCR mix, (Invitrogen). The RT-qPCR amplification used the following thermal cycling conditions: 15 min at 42–50°C and 2 min at 95°C followed by 45 cycles of 15 s at 95°C and 30–60 s at 60°C. Each reaction mixture contained 800 nmoles of each primer and 250 nmoles of probe.

Samples with Ct values below 40 were considered positive and values 40 to 45 were considered suspicious.

Between 2004 and 2011, pooled samples were used for influenza A RT-qPCR results and reports. Each pool sample comprised of three 500 μL field samples of the same species collected at the same time and the same location. Positive influenza A RT-qPCR pool samples were tested individually for H5 and H7. From 2012 onwards, individual samples were always tested and reported. A bird was considered positive if either or both of the swabs (cloacal and/or oropharyngeal) were positive (Table 1 and S1 Table). Most penguin and shearwater samples were tested individually.

### Virus isolation and determination of subtype and pathotype

Virus isolation was conducted for all samples that were positive for H5 or H7 by the H5/H7- specific TaqMan RT-qPCRs (Table 1) in addition to 15% randomly selected samples (with Ct values 20–39) that were negative by H5/H7- specific TaqMan RT-qPCRs but positive by influenza A RT-qPCR. Swab samples were inoculated into the allantoic cavity of 9- to 10-day-old embryonated specific-pathogen-free chicken eggs, using established procedures, and were checked for hemagglutinating activity [21].

All allantoic fluid samples that tested positive by the hemagglutination test were tested by influenza A RT-qPCR [10] to confirm the presence of AIV. Hemagglutinin (HA) subtyping of all isolated AIVs was conducted using the HI test [21], using reference antisera to all 16 known subtypes, and was confirmed by influenza A–specific HA RT-PCR and partial amplicon sequencing [22, 23]. Neuraminidase (NA) subtyping was performed by RT-PCR, using NA universal primers, followed by sequencing as described previously [23] (Table 2).

**Table 2:** HA and NA subtypes for 326 AIV isolates from wild bird surveillance in New Zealand, 2004–2020. Virus isolation was conducted on all samples that tested positive by specific H5 or H7 RT-qPCR and on 10%–15% of samples that tested positive by influenza A matrix gene RT-qPCR per location. (In total, 326 AIVs were isolated, although the exact HA/NA combinations could be determined for only 306 isolates).

HA NA	H1	H2	H3	H4	H5	H6	H7	H8	H9	H10	H11	H12
**N1**	6		9		5	1				1	1	
**N2**	5		16	9	6	9					4	
**N3**		3	1	2	6					15	4	
**N4**										2		
**N5**							1			2		1
**N6**			6	99		2				1		
**N7**		1		2	1		9			7		
**N8**			52	3	1	2			1			
**N9**		1		2	1	1					5	
**N?**	3		6	8							4	

The HA cleavage site of all H5 and H7 isolates was determined by sequencing with RT-PCR primers [23, 24]. RT-PCR was performed using a 25-μL volume (20 μL of RT-PCR mix and 5 μL of nucleic acid) and a SuperScript III One-Step RT-PCR System with Platinum *Taq* mix (Invitrogen). The RT-PCR amplification used the following temperature cycle: 15–30 min at 50°C and 2 min at 94°C, followed by 35 cycles of 30 s at 94°C, 45 s at 60°C, and 90–180 s at 68°C, with a final elongation step at 68°C for 5 min.

### Phylogeny of avian influenza virus H5 and H7 HA genes

#### Dataset preparation

H5 and H7 HA avian influenza nucleotide datasets were compiled by downloading samples from the GenBank influenza database. New Zealand sequences for the H5 subtype have the GenBank accession numbers MH230000, MH231588, MH236436, MH236437, MH244442, MH244443, MH244444, MH276959, MH276986, OP776660, and CY014640. For the H7 subtype, the New Zealand sequences have the accession numbers CY061618, MH276960, MH276961, MH276981, MH276987, and OP874597. The full set consists of complete sequences from all geographic locations (7485 H5 sequences, 3051 H7 sequences) and both partial and complete sequences from Australia (40 H5 sequences, 42 H7 sequences) and New Zealand (11 H5 sequences, 6 H7 sequences). Sequences lacking the date or country of origin were excluded.

BLASTN [25] was used to identify possible homologous sequences, with New Zealand sequences being used as the query. Sequences from non-avian hosts and sequences without country information were filtered out of the BLASTN results. To prepare datasets for Bayesian analysis, samples were taken from the full set. All sequences from New Zealand, Australia, or countries matched by BLASTN were included, and non-matching countries were subsampled at 10% per country. Countries matched by BLASTN are shown in the S2 and S3 Tables. Sample collection dates were used for time calibration in the analyses. For sample dates missing month or day information, the middle date of that year or month was used for calibration purposes.

Multiple sequence alignment was performed using MAFFT v7.52 [26] with the default parameter settings. To test for temporal signals and to remove outliers, we used TempEst v1.5.3 [27] and IQ-Tree [28] with a GTR substitution model [29] and gamma rate heterogeneity [30]. Three outliers were detected by TempEst in the H5 dataset (LC387332, MW334543, and MH598105), and these were excluded from our analyses. Our datasets for Bayesian analysis have a total of 2147 sequences for the H5 subtype and 2723 sequences for the H7 subtype.

### Bayesian models

Analyses were performed using BEAST 2.6 [31, 32], using an HKY substitution model [33] with a Dirichlet prior and concentration α = (3,3,3,3) on the nucleotide frequencies, gamma rate heterogeneity with four rate categories, a strict clock with an exponential clock rate prior with a mean of 3∙10^−3^, and a Skyline coalescent model [34] with a log normal population size prior with mean = 1 and standard deviation = 2. The clock rate prior was chosen to have a broad 95^th^-percentile range from 2.5∙10^−5^ to 3.7∙10^−3^, based on the average substitution rates for AIV of 1∙10^−3^ to 3∙10^−3^ reported in previous studies [35–37]. Monte Carlo Markov chains of length 100 million were run.

### Serology—H5 and H7 HI testing

Collected mallard duck sera were screened for H5 and H7 antibodies by using an HI test and the method described [21], using inactivated H5N2, H5N3, H7N3, and H7N7 antigens. Results were recorded for all samples with titers of ≥1:4 (Table 3).

**Table 3:** Serological surveillance for avian influenza virus (AIV) H5 and H7 in mallards in New Zealand, 2004–2007.

Year of sampling	Species	No. of birds (sera) tested	No. of sera with HI titers between ≥1/4 and 1:256, using two H5 and H7 HI antigens*			
			H5N2	H5N3	H7N3	H7N7
2004	Mallard and paradise ducks	215	16	16	4	0
2005	Mallard	257	72	32	18	4
2006	Mallard	108	15	n/t	2	n/t
2007	Mallard	247	29	10	1	n/t
**Total**		**827**	**132**	**58**	**25**	**4**

n/t = not tested. The following AIVs were used for preparing HI antigens: A/mallard/NZ/1/97 for H5N2; A/tern/Australia/75 for H5N3; A/chicken/VIC/224/92 for H7N3; and A/mallard/NZ/1365-355/06 for H7N7.

### Statistical analysis

Statistical analysis was carried out on testing data for mallard ducks sampled between 2012 and 2020 to avoid pooled samples collected before 2012. During this period, sampling was undertaken at 18 different locations across New Zealand. The findings are presented at the level of the territorial authority (TLA [district]; see S1 Fig) to preserve privacy (precise sampling locations within the TLA varied from year to year but were in proximity (<25 km apart). Only TLAs with three or more sampling events were included in the analysis. The resulting four TLA groupings represented a total of 10 locations: three in the Gisborne District, one in the Western Bay of Plenty District, one in the Hastings District, and five in the Hauraki District (see Fig 1). Logistic regression was applied to investigate the association of AIV infection with different risk factors. The AIV status of an individual bird was the outcome variable, and the explanatory variables were the year, TLA, and region. First, the influence of individual risk factors on AIV status was examined through univariate logistic regression analysis, and their significance with respect to the presence of AIV infection was assessed, with a screening threshold *P* value of <0.2, based on the likelihood-ratio test (LRT). All the risk factors were found to be significant. Because of collinearity among the location variables, the region was excluded from the multivariate analysis.

For the multivariate logistic regression, the year and sampling location were considered as risk factors, i.e., predictor variables that had a *P* value of less than 0.05 by the LRT and were retained in the model.

A two-way interaction between year and sampling location was examined and was included in the model if it was significant (*P* < 0.05) and if an improvement in the Akaike information criterion (AIC) of the model was observed. Goodness of fit measures were used to evaluate the performance of the final model (Table 1).

Multivariate logistic regression analyses with AIV H5 and H7 status (separately) as the outcome variable were also performed, following the same process.

All analyses were conducted in R version 4.2.2 [38–42].

## Results

### Birds sampled

From 2004 to 2009, samples were collected from 1269 migratory shorebirds. Cloacal swabs were collected from 975 red knots, 280 bar-tailed godwits, and 14 ruddy turnstones. In addition, oropharyngeal swabs were collected from 314 knots and 125 godwits (Table 1). Most of these samples were collected at Pūkorokoro Miranda on the Firth of Thames in the North Island, but samples were also collected from birds at Kaipara Harbour (n  =  14) in the Northland region and in Motueka (n  =  6) in the Nelson Marlborough region of the South Island (Fig 2).

From 2004 to 2020, samples were collected from 15,740 mallards (*Anas platyrhynchos*) 96 paradise shelducks (*Tadorna variegata*), and 50 grey teal (*Anas gracilis*). In addition, from 2009 to 2010, cloacal and oropharyngeal swabs were collected from 835 black-billed gulls (*Chroicocephalus bulleri*), 318 black-backed gulls (*Larus dominicanus*), 109 little blue penguins (*Eudyptula minor*), 58 yellow-eyed penguins (*Megadyptes antipodes*), 80 wrybills (*Anarhynchus frontalis*), and 80 sooty shearwaters (*Ardenna grisea*) (Table 1). Between 2004 and 2007, blood samples were also collected from 827 mallards and paradise ducks [S1 Table].

### AIV in migratory shorebirds in New Zealand

In 2006, AIV RNA was detected by influenza A RT-qPCR in two cloacal samples collected from red knots and its identity was confirmed using nested matrix PCR. When the matrix gene amplification products (740 bp) were sequenced and compared to sequences deposited in GenBank, they had the highest identity (96.5%) with the matrix gene sequences of AIV A/chicken/Hubei/wk/1997(H5N1) (DQ997124). No virus was isolated from these samples, making further characterization impossible.

### AIVs in waterfowl in New Zealand

Over the 17 years of targeted surveillance, the only samples from resident waterfowl that tested positive for AIV (by RT-qPCR or virus isolation) were collected from mallards or grey teal. A total of 326 AIVs with 41 subtypes were isolated from mallards (Tables 1 and 2). No viruses were isolated from grey teal. Of the 15,740 mallards tested by influenza A RT-qPCR, 6179 (43.7%) were classified as positive for AIV RNA (by oropharyngeal swabs, cloacal swabs, or both). However, because samples were pooled before 2012, the true prevalence of infected birds (50.5%) could be calculated only during the last 8 years, when individual samples were tested. RT-qPCR testing for H5 and H7 subtypes was undertaken at the individual bird level for the entire study period (2004–2020).

**Fig 3:**
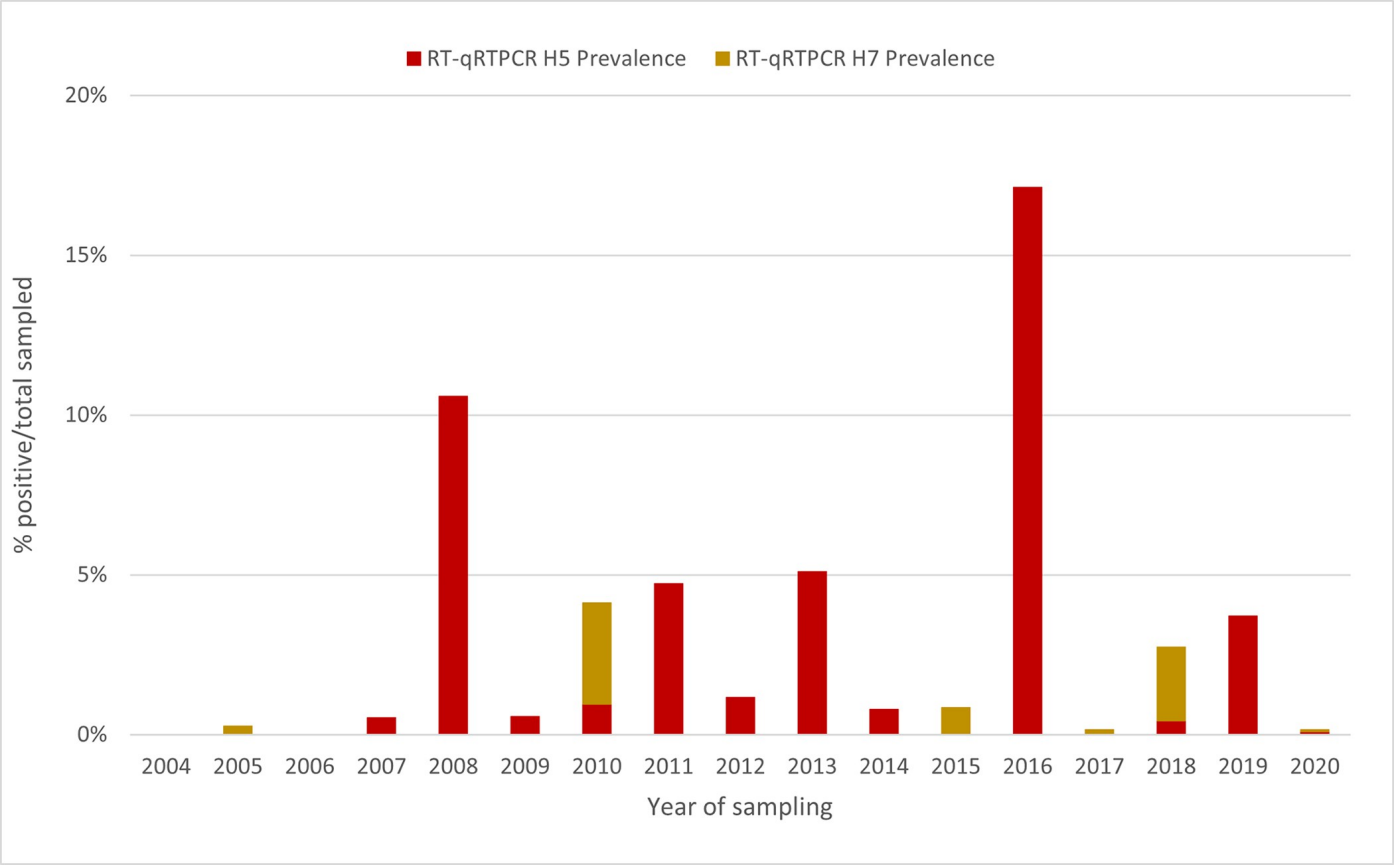
Prevalence of H5 and H7 AIVs as determined by RT-qPCR in mallard ducks in New Zealand, 2004–2020.

**Fig 4:**
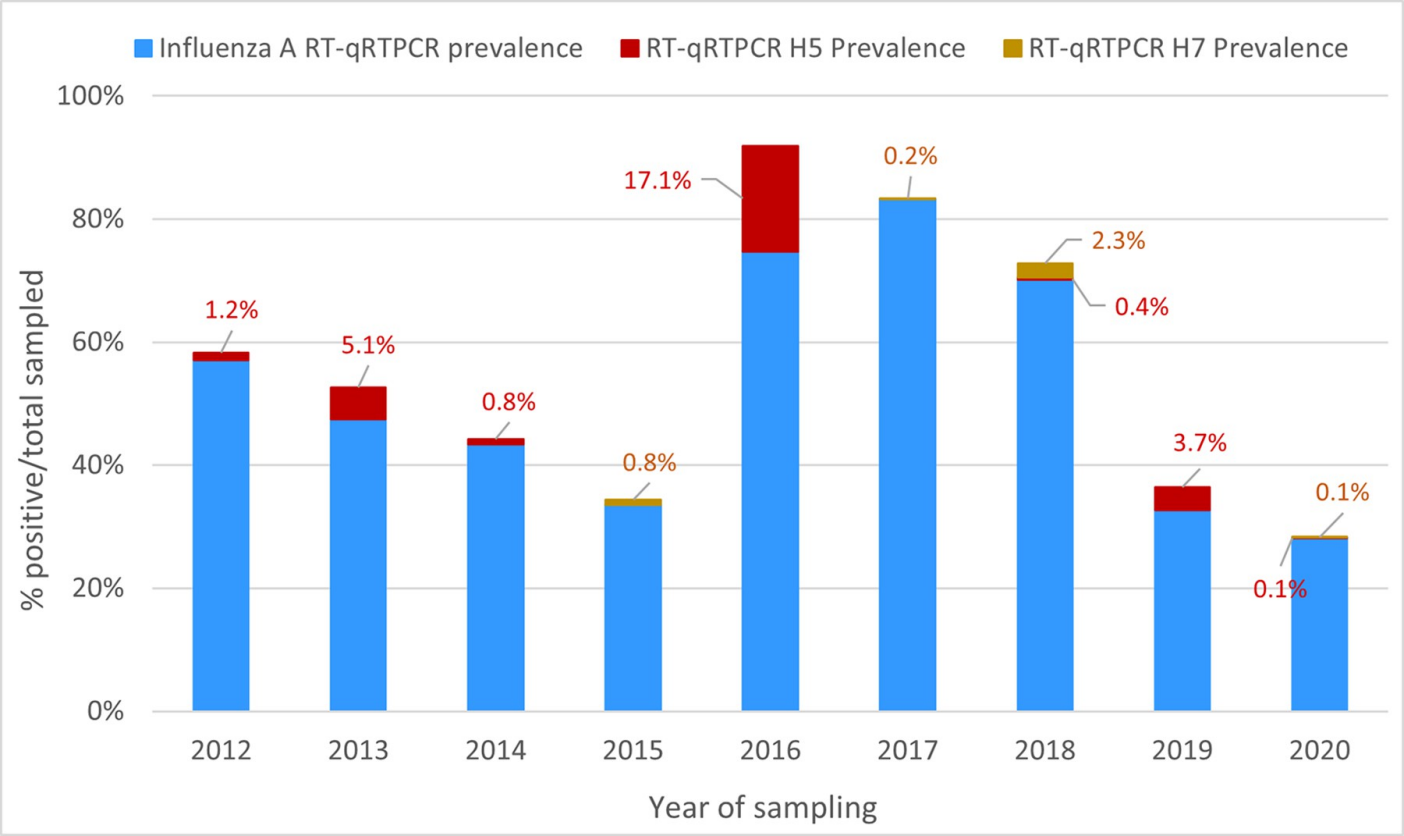
Prevalence of influenza A, H5, and H7 genes as detected by RT-qPCR in mallard ducks in New Zealand, 2012–2020.

The H5 viruses confirmed by RT-qPCR during this period were detected in 12 of the 17 years of the study period, whereas H7 viruses were detected in only six of the 17 years (Fig 3). The overall prevalence of detected RNA was 1.8% (344 birds) for H5 viruses and 0.3% (51 birds) for H7 viruses. In the years with positive test results, the prevalence in sampled birds ranged from 0.2% to 17% for H5 viruses and from 0.1% to 2% for H7 viruses (Table 1 and Figs 3 and 4). Virus was isolated successfully from samples with Ct values below 30 but only very inconsistently from samples with Ct values above 30.

### Summary tree for the H5-subtype HA gene derived from Bayesian analysis using a HKY substitution model, a strict clock, and a constant coalescent model

The H5 dataset contained 2147 sequences and 2056 locations where samples were collected globally between 1966 and 2023. Three sequences were detected as outliers by TempEst and removed. The outliers detected were LC387332 from Vietnam (from a sample dated 2017/07/18), MW334543 from Taiwan (from a sample dated 2018/02/12), and MH598105 from the USA (from a sample dated 2001/05/23).

**Fig 5:**
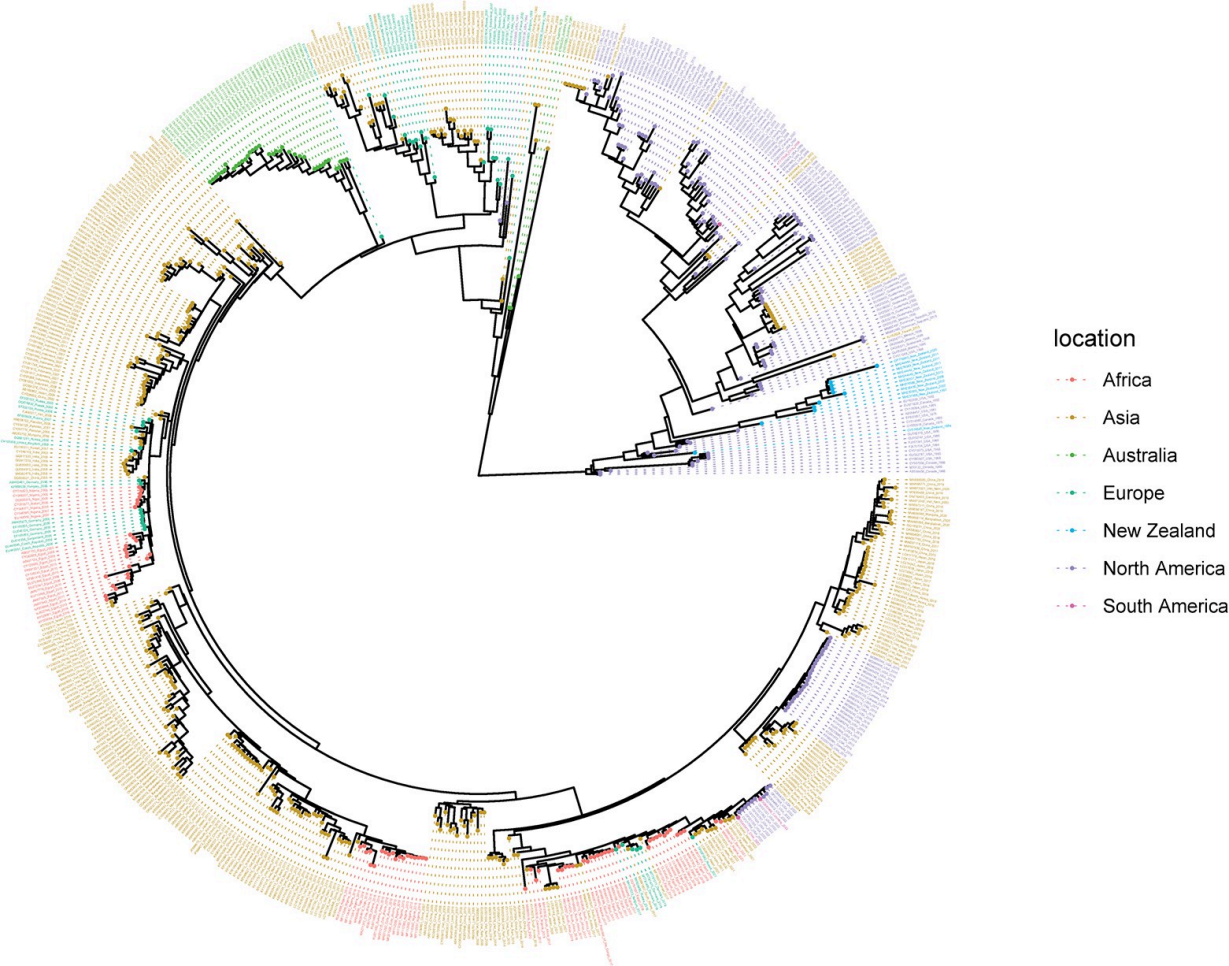
Summary tree for the H5 subtype derived from Bayesian analysis using a HKY substitution model, a strict clock, and a constant coalescent model. Maximum clade credibility was used to construct the summary tree. The sequences are coloured according to the sampling location, i.e., Asia (yellow), Africa (red), Europe (dark green), North America (purple), South America (pink), Australia (lime green), or New Zealand (blue). Sequences with high similarity from the same country are down-sampled for readability.

All but one of the New Zealand H5 sequences formed a monophyletic clade. The exception was the oldest New Zealand sequence (CY014640, from the year 1984), which fell within the larger North American sister clade (Fig 5). These data suggest that there has been only limited introduction of H5 viruses into New Zealand and long-term circulation of one lineage within resident birds. The Australian sequences form two clades, both within the Eurasian clade, that are distinct from the New Zealand sequences.

### Time-structured tree

**Fig 6:**
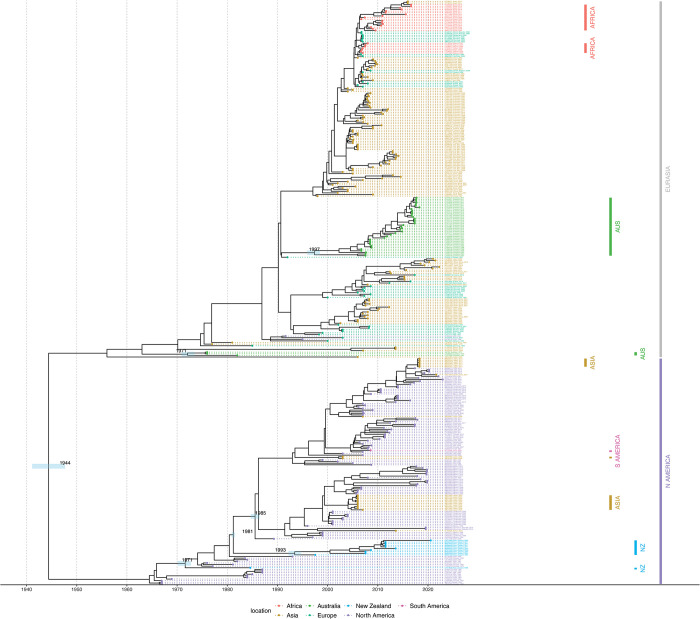
Timed phylogeny of the summary tree for the H5 subtype, using an HKY substitution model, a strict clock, and a constant coalescent model. The 95% highest posterior density estimates of the most recent common ancestors (MRCAs) for the New Zealand clade, New Zealand’s sister clade (the North American clade), and the Australian clade are shown as bars. The time scale is in years. Sequences with high similarity from the same country are down-sampled, and the most distant clade (the Eurasian/African/North American clade) is collapsed for improved readability.

The timed summary tree is shown in Fig 6, with the time shown in calendar years. The 95% posterior interval for the estimated root age of the tree from the sampled sequences is from the 1940s to the 1950s. The mean estimated age of the most recent common ancestor (MRCA) for the New Zealand clade (excluding the oldest sample, CY014640) is 1993, and the mean estimated age when all New Zealand samples are included is 1971. The mean estimated age of the MRCA of the North American sister clade is 1985.

### Summary tree for the H7 subtype HA gene from Bayesian analysis using an HKY substitution model, a strict clock, and a constant coalescent model

**Fig 7:**
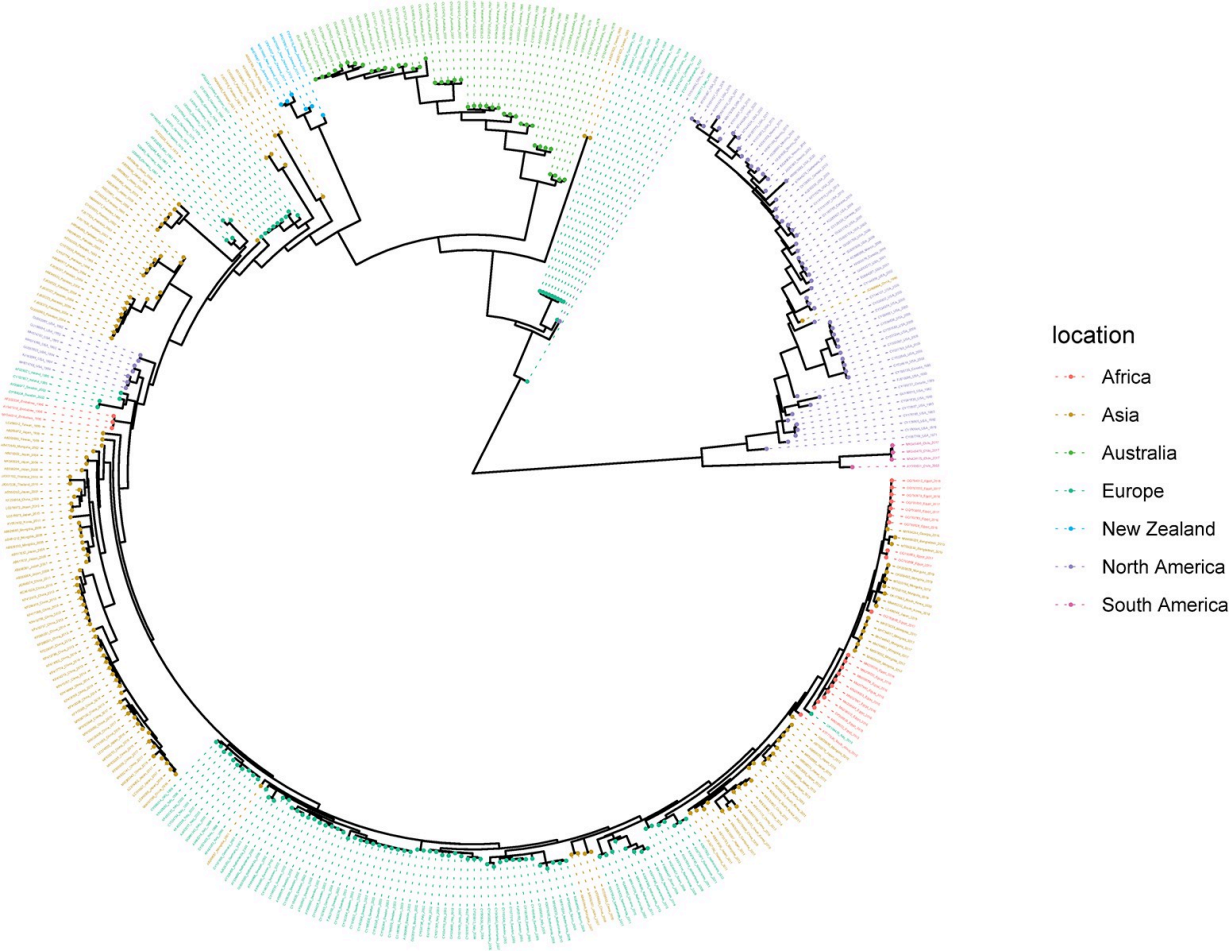
Summary tree for the H7 subtype from Bayesian analysis using an HKY substitution model, a strict clock, and a constant coalescent model. Maximum clade credibility was used to construct the summary tree. The sequences are colored by sampling location, i.e., Asia (yellow), Africa (red), Europe (dark green), North America (purple), South America (pink), Australia (lime green), or New Zealand (blue). Sequences with high similarity from the same country are down-sampled for readability.

**Fig 8:**
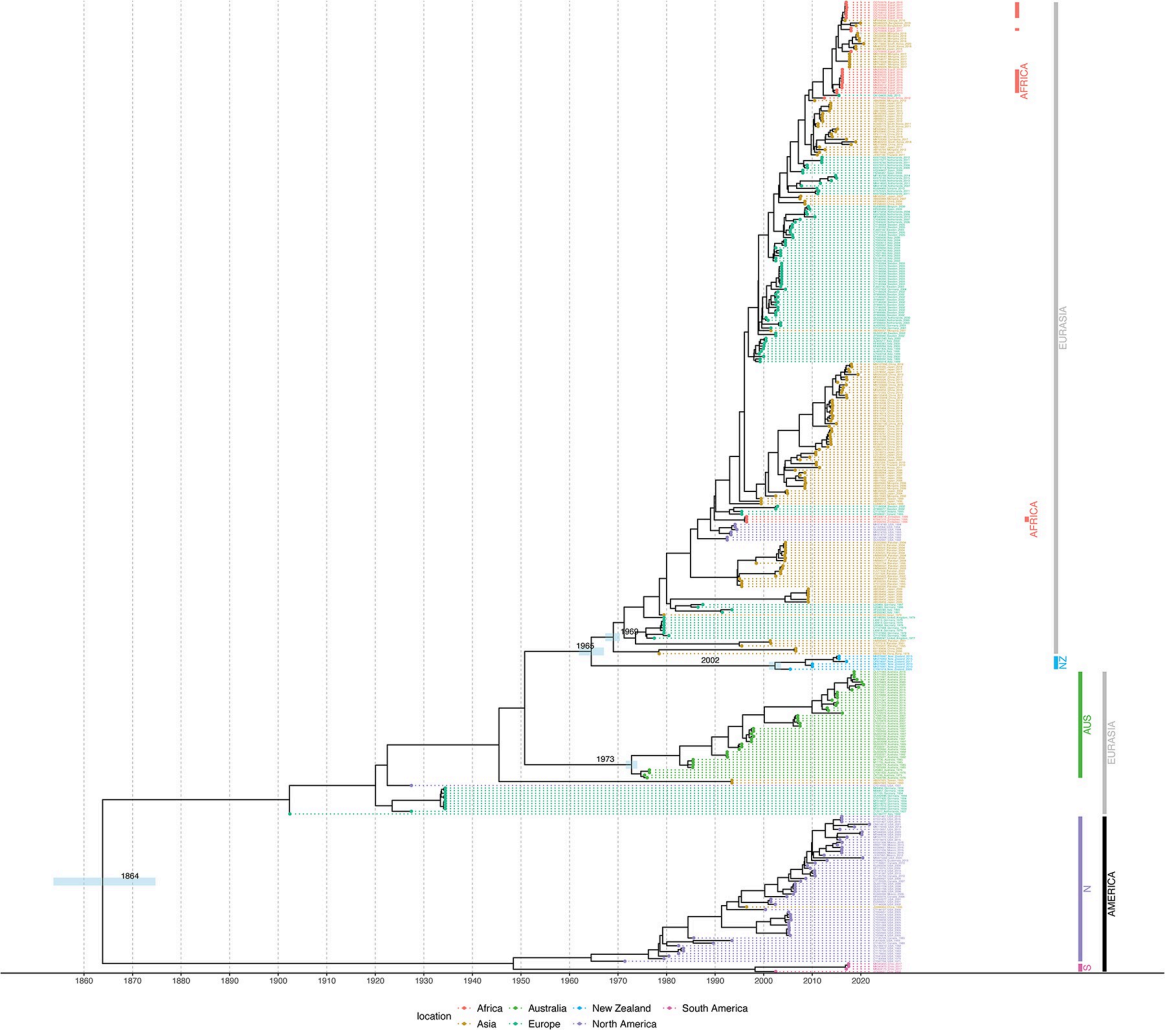
Timed phylogeny of the summary tree for the H7 subtype HA gene, using an HKY substitution model, a strict clock, and a constant coalescent model. The 95% highest posterior density estimates of the most recent common ancestors for the New Zealand clade, New Zealand’s sister clade (the Eurasian clade), and the Australian clade are shown as bars. The time scale is in years. Sequences with high similarity from the same country are down-sampled for readability.

The H7 dataset contained 2723 sequences and 1865 locations where samples were collected globally between 1902 and 2021. Fig 7 shows the summary tree colored by sampling location. As with the H5 sequences, all of the H7 New Zealand sequences form a monophyletic clade within a wider clade containing mostly Eurasian sequences, which is again suggestive of there having been only limited introduction of viruses (Fig 7). The Australian sequences form a monophyletic clade as an outgroup to the New Zealand and Eurasian clade. Fig 8 shows the timed phylogeny for the summary tree. The estimated mean age of the MRCA of the New Zealand samples is 2002. The MRCA of the sister Eurasian-like clade has a mean age of 1969. The MRCA of the Australian clade has an estimated mean age of 1973.

### Serological HI testing

A total of 132 of 827 sera collected (16%) reacted with an H5 HI antigen with titers ranging between 1:4 and 1:128, and 25 of 827 sera (3.0%) reacted with H7 HI antigen with titers between 1:4 and 1:32 (Table 3).

### Logistic regression

The results of the multivariate logistic regression considering the association between sampling location, sampling year, and the AIV status of an individual bird are reported in Table 4. Both sampling year and sampling location had statistically significant effects on the AIV status of an individual bird.

**Table 4:** Multivariate analysis of the association between explanatory variables and AIV infection in individual mallards tested in New Zealand from 2012 to 2020.

Variable	Category	Estimate	SE	OR (95% CI)	*P*	*P* (LRT)*
Year	2012 (reference)					** **
	2013	−0.17	0.19	0.84 (0.58–1.22)	0.36	<0.0001
	2014	1.29	0.26	3.62 (2.22–6.08)	0.00	
	2015	–1.39	0.35	0.25 (0.13–0.50)	0.00	
	2016	1.86	0.35	6.44 (3.27–13.16)	0.00	
	2017	0.62	0.33	1.87 (1.00–3.61)	0.06	
	2018	1.14	0.38	3.14 (1.52–6.71)	0.00	
	2019	–2.06	0.18	0.13 (0.09–0.18)	0.00	
	2020	–1.44	0.18	0.24 (0.17–0.33)	0.00	
Sampling location	Gisborne District (reference)					
	Hauraki Plains	0.55	0.34	1.73 (0.91–3.40)	0.10	<0.0001
	Lake Te Rotokare	–0.40	0.34	0.67 (0.35–1.31)	0.23	
	Mouth of Kaituna River	1.72	0.27	5.61 (3.36–9.85)	0.00	
Year*sampling location	2013—Hauraki Plains	–0.46	0.27	0.63 (0.38–1.06)	0.08	
	2014—Hauraki Plains	–2.50	0.32	0.08 (0.04–0.15)	0.00	<0.0001
	2015—Hauraki Plains	–0.02	0.40	0.98 (0.44–2.11)	0.96	
	2016—Hauraki Plains	0.92	0.46	2.51 (1.03–6.14)	0.04	
	2018—Hauraki Plains	–0.62	0.25	0.54 (0.23–1.21)	0.00	
	2019—Hauraki Plains	1.83	0.25	6.21 (3.78–10.26)	0.00	
	2020—Hauraki Plains	0.89	0.27	2.44 (1.49–4.01)	0.03	
	2013—Lake Te Rotokare	0.59	0.34	1.80 (1.06–3.07)	0.00	
	2014—Lake Te Rotokare	–2.04	0.42	0.13 (0.07–0.25)	0.14	
	2016—Lake Te Rotokare	–0.62	0.43	0.54 (0.23–1.22)	0.00	
	2018—Lake Te Rotokare	–1.65	0.29	0.19 (0.08–0.44)	0.66	
	2019—Lake Te Rotokare	2.76	0.34	15.84 (9.09–27.77)	0.21	
	2020—Lake Te Rotokare	0.13	0.35	1.14 (0.64–2.00)	0.00	
	2015—Mouth of Kaituna River	0.43	0.30	1.53 (0.78–2.94)	0.14	
	2016—Mouth of Kaituna River	–2.61	0.19	0.07 (0.04–0.14)	0.36	
	2017—Mouth of Kaituna River	–0.26	0.26	0.77(0.40–1.47)	0.00	
	2018—Mouth of Kaituna River	–2.09	0.35	0.12 (0.06–0.25)	0.00	

*LRT = likelihood-ratio test.

A two-way interaction between year and sampling location was significant, and an improvement in the AIC of the model was observed. The interaction term was included in the final model.

The Pseudo R^2^ (McFadden) value of the model was 0.2142, and the area under the receiver operating characteristics (ROC) curve was 0.7943, showing that the model had good discriminatory ability in terms of predicting AIV status. Checking for overdispersion, we found the deviance to be 1.093. As the value was close to 1, there was no overdispersion on the prediction model.

The models developed for infection with H5 subtypes and H7 subtypes found no interaction between the exploratory variables, and only the main effects of year and sampling location were included in the model. Although the goodness of fit measures showed the models to be apparently well fitted, we cannot draw firm conclusions because of several coefficients with confidence intervals going to infinity; this is probably a consequence of the small number of cases. The results obtained with the models are shown in the S4–S8 Tables.

## Discussion

To better understand the potential risk of HPAI virus being introduced by migratory birds into New Zealand, the Ministry for Primary Industries (previously MAF) enhanced its wild bird AIV surveillance activities starting in 2004 which have been sustained to date. The results of this active surveillance activity are summarized here and represent the largest such study to date in New Zealand. Of particular importance to New Zealand, outside of the risks posed to farmed poultry species, is the risk to native wild birds, given the threatened status of many of New Zealand’s unique avian species, such as the kiwi (*Apteryx* spp.), takahe (*Porphyrio hochstetteri*), Campbell Island teal (*Anas nesiotis*), black robin (*Petroica traversi*), black stilt (*Himantopus novaezelandiae*), and New Zealand storm petrel (*Fregetta maoriana*). The findings of this study provide valuable baseline data that can be used for future risk assessment and preparedness planning.

This study found limited evidence for AIV carriage in arriving northern hemisphere migrant birds. We were unable to isolate any AIVs from shorebirds upon their arrival, suggesting that few viable viruses are carried to New Zealand by this route [43–45]. Nevertheless, two samples from red knots tested positive for AIV RNA. Although virus could not be isolated from these samples, we were able to generate sequences from the matrix gene RNA segment, but not from the HA/NA genes. Therefore, it was possible to derive only limited genomic information relating to the epidemiology of the virus. The ecology and epidemiology of AIV are not as well understood in shorebirds as they are in waterfowl. In the United States, sampling has focused on the unique ecosystem of the Delaware Bay region of the East Coast in spring. AIV subtypes such as H3, H10, and H11 are regularly isolated, whereas H1, H2, H4, H5, H6, H7, H9, and H12 are less frequently isolated [46, 47]. In Australia, a recent study by Wille et al. (2023) detected AIV positivity and seroconversion, especially in red knots and ruddy turnstones [48]. This is in contrast with the general absence of AIV in shorebirds in Europe [49] (with some exceptions [50]). Our New Zealand results are consistent with other studies of recent shorebird arrivals, indicating that migratory shorebirds in general do not persistently maintain AIVs during long-distance migration [43, 44, 51]. Because of the difficulties associated with sampling newly arrived migrant birds, the sample sizes were small and the prevalence was possibly below the detectable threshold in birds sampled in the East Asian–Australasian Flyway [43, 44, 50, 52]. AIVs have rarely been isolated from shorebirds in this flyway [52]; however, Chen et al. [53] reported finding seropositive shorebirds on their southbound migration near Shanghai. Even if such birds were infected before departing Alaska, it remains uncertain whether infected birds could complete an 11,000-km non-stop flight to New Zealand—LPAI infection of swans in Europe apparently reduced their migratory performance [54]—or maintain a virus within a migratory population for so long. Red knots that have stopovers along the eastern seaboard areas of China, South-East Asia, and Australia pose a different risk as they can potentially become infected with exotic influenza viruses while traveling to New Zealand.

In contrast to the situation in migratory shorebirds, we found evidence that AIV are widely circulating in resident mallards in New Zealand. The prevalence of AIV in mallards, as determined by influenza A RT-qPCR, could not be determined for 2004–2011 because only pooled samples were tested. However, from 2012 to 2020, individual birds were tested and an AIV annual prevalence between 28.2% and 83.2% was observed for this period (Table 1, Figs 3 and 4). Of relevance is the mid-summer timing of our sampling events, which coincides with the highest prevalence of juvenile ducks. Juvenile ducks are immunologically naïve and are more susceptible than adults to AIV infection, which may explain the high prevalence of AIV in this study. This latter finding is consistent with the results of surveillance studies in other parts of the world [8, 45, 55, 56], although Australian studies have found a much lower prevalence [37, 38]. The differences in AIV prevalence may be attributed to several factors including the species sampled, the age of the birds, the timing/season, and the testing and sample collection procedures. In our study, most ducks were trapped in walk-in funnel traps overnight and the samples were not collected until the next morning. Therefore, cross-infection/contamination could have occurred during the interval between trapping and sample collection.

From a random selection of samples that tested positive by influenza A RT-qPCR but were negative in H5 and H7 RT-qPCR, the most commonly isolated AIV HA groups were H4 (38.6%), H3 (27.6%), and H10 (8.6%). Subtypes H8, H13, H14, H15, and H16 were not isolated from wild ducks in this study. The NA subtypes isolated most often were N6 (33.3%), N8 (17.9%), and N2 (14.8%) (Table 2). In a comparable study in North America, the predominant HA subtypes isolated from wild ducks were H6, H3 and H4, whereas the most common NA subtypes detected were N8, N2 and N6 [8, 45, 55, 56]. We were able to isolate only a few viruses from samples positive for H5 and H7 by RT-qPCR, most likely because of a low amount (or lack) of viable virus in the original sample, variable fitness of different strains with respect to growth in embryonated eggs, or the potential for competition in mixed infections [57].

The phylogenetic analysis of the HA gene of the New Zealand H5 and H7 waterfowl influenza viruses shows that they primarily cluster together within each subtype. This suggests that these viruses have circulated for decades within New Zealand, with no evidence of recent introductions. The viruses appear to be the result of a limited number of introductions, consistent with the resident nature of mallards in New Zealand and with our low rate of AIV detection in shorebirds. New Zealand is not on a migratory flyway for waterfowl in general and, hence, lacks the exposure to migratory species that commonly contribute to AIV spread around the world. The isolated evolution of AIV in New Zealand identified through this study is consistent with the findings of comparable studies in Australia [43, 44, 58]. Of note, the H5 HA gene viruses are phylogenetically closer to the LPAI North American H5 HA wild bird viruses whereas H7 HA viruses form a sister cluster relationship with wild bird viruses of the Eurasian and Australian lineages. A further finding of our study, supporting New Zealand’s geographic isolation, is the distant relationship between Australian and New Zealand H5 subtypes.

The HI titers obtained when sera from waterfowl were tested with H5 and H7 antigens provided additional evidence of the presence of these subtypes in New Zealand’s resident mallard population. Serological data proved useful in those years when no viruses were isolated/detected (e.g., 2004, 2005, and 2006 for the H5 subtype and 2004, 2006, and 2007 for the H7 subtype). However, cross-reactivity between influenza NA antigens can complicate the interpretation of this testing. Therefore, using two H5 and H7 antigens, each with a different NA (as in this study) helped reduce any potential cross-reactivity. Further considerations when using serology include the fact that low titers may not be detected if an antigenically related subtype is not used [59] as well as animal welfare concerns, given that blood collection can be time consuming and stressful for birds. Serology is, therefore, generally recommended only in specific circumstances.

The multivariate analysis showed that year and sampling location had statistically significant effects on the occurrence of AIV in New Zealand. However, the AIV risk of a site was not consistent year-on-year, which makes predicting circulating AIV in wild birds very difficult. This highlights the importance of the national-level passive surveillance for the early detection of exotic HPAI viruses in wild birds, poultry, and other susceptible species. Our longitudinal study demonstrates the importance of ongoing work to provide a sound understanding of the epidemiology, evolution, and ecology of AIVs in the New Zealand context.

Overall, our data presented support the view that, although HPAI (H5 and H7) viruses can potentially enter and become established in New Zealand via the East Asian–Australian migratory flyway, the risk of this occurring has historically been very low.

Our study findings of endemic LPAI circulating in New Zealand waterfowl support recommendations that minimize direct and indirect contact between resident waterfowl and commercial poultry through enhanced biosecurity to prevent the introduction of AIV into domestic poultry flocks. Our study shows it is important to continue to understand currently circulating AIVs in bird populations in New Zealand, as doing so increases the confidence of detecting novel introductions and can inform surveillance design and control measures for AIVs.

## Supporting information

S1 Fig Odds ratios determined through multivariate analysis of associations between each risk factors and AIV infection from surveillance data obtained in New Zealand from 2012 to 2020. (TIF)

S2 Fig Importance of risk factors in multivariate analysis of associations between each risk factor and AIV infection from surveillance data obtained in New Zealand from 2012 to 2020. (TIF)

S3 Fig Receiver operating curve for avian influenza subtype H5 from surveillance data obtained in New Zealand from 2012 to 2020. (TIF)

S4 Fig Receiver operating curve for Avian influenza subtype H7 from surveillance data obtained in New Zealand from 2012 to 2020. (TIF)

S1 Table Date and location of sampling, species, and other information for birds sampled from 2004 to 2020. (DOCX)

S2 Table Summary of BLASTN hits for New Zealand query sequences of the H5 subtype. (DOCX)

S3 Table Summary of BLASTN hits for New Zealand query sequences of the H7 subtype. (DOCX)

S4 Table Models fitted to explain AIV infection in mallard ducks sampled in New Zealand from 2012 to 2020. (DOCX)

S5 Table Univariate analysis of association between year and AIV infection from surveillance data obtained in New Zealand from 2012 to 2020. (DOCX)

S6 Table Univariate analysis of association between territorial authority and AIV infection from surveillance data obtained in New Zealand from 2012 to 2020. (DOCX)

S7 Table Multivariate analysis of associations between each risk factors and H5 subtype from surveillance data in New Zealand from 2012 to 2020. (DOCX)

S8 Table Multivariate analysis of associations between each risk factors and H7 subtype from surveillance data obtained in New Zealand from 2012 to 2020. (DOCX)
