# Supplementary figures and images for: Avian influenza viruses in New Zealand wild birds, with an emphasis on subtypes H5 and H7: Their distinctive epidemiology and genomic properties
Source: PLoS One. 2024 Jun 3;19(6):e0303756. doi: 10.1371/journal.pone.0303756 (PMC11146706; doi:10.1371/journal.pone.0303756)

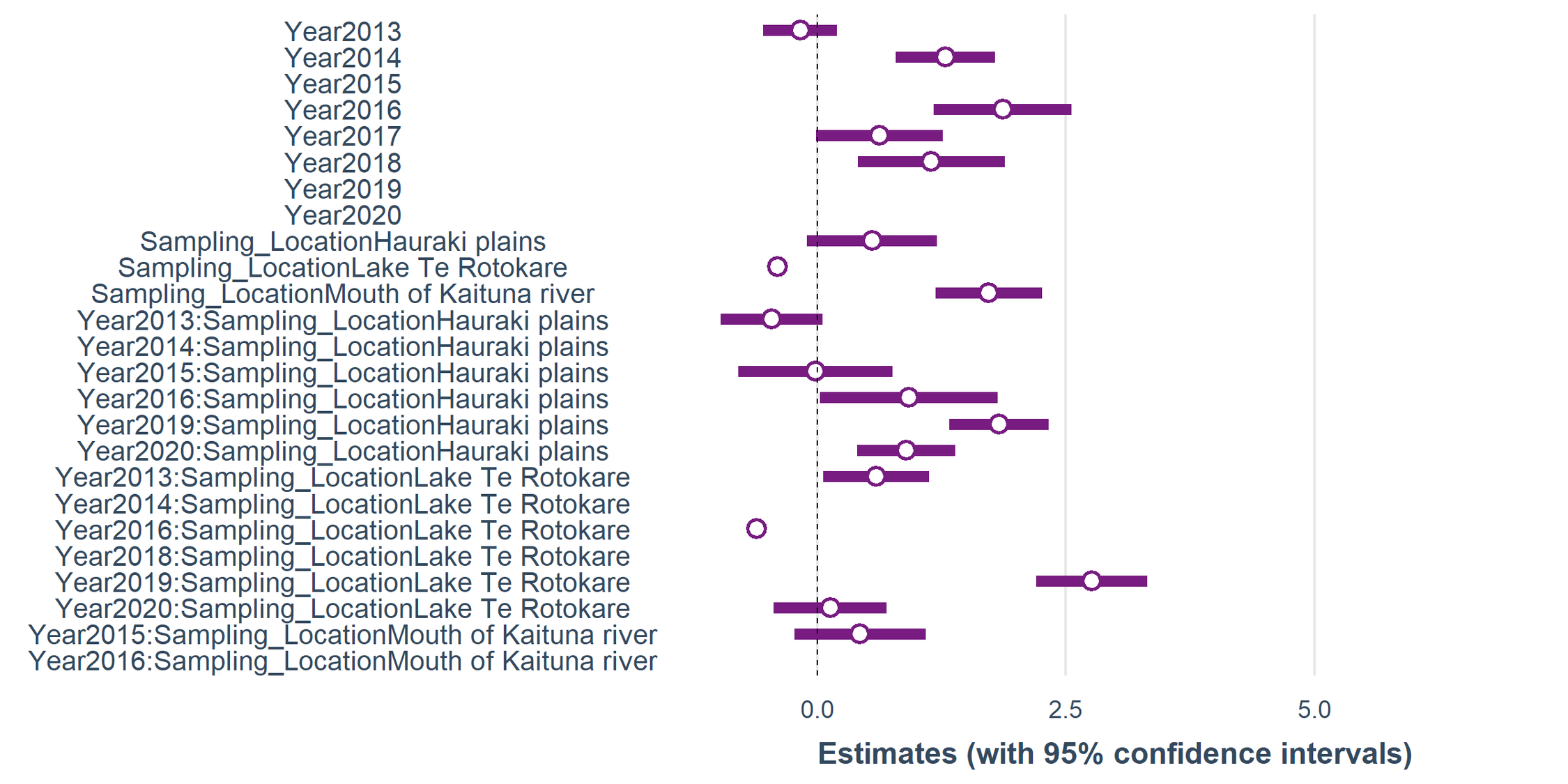

Supplement: S1 Fig — (TIF) [file pone.0303756.s001.tif]

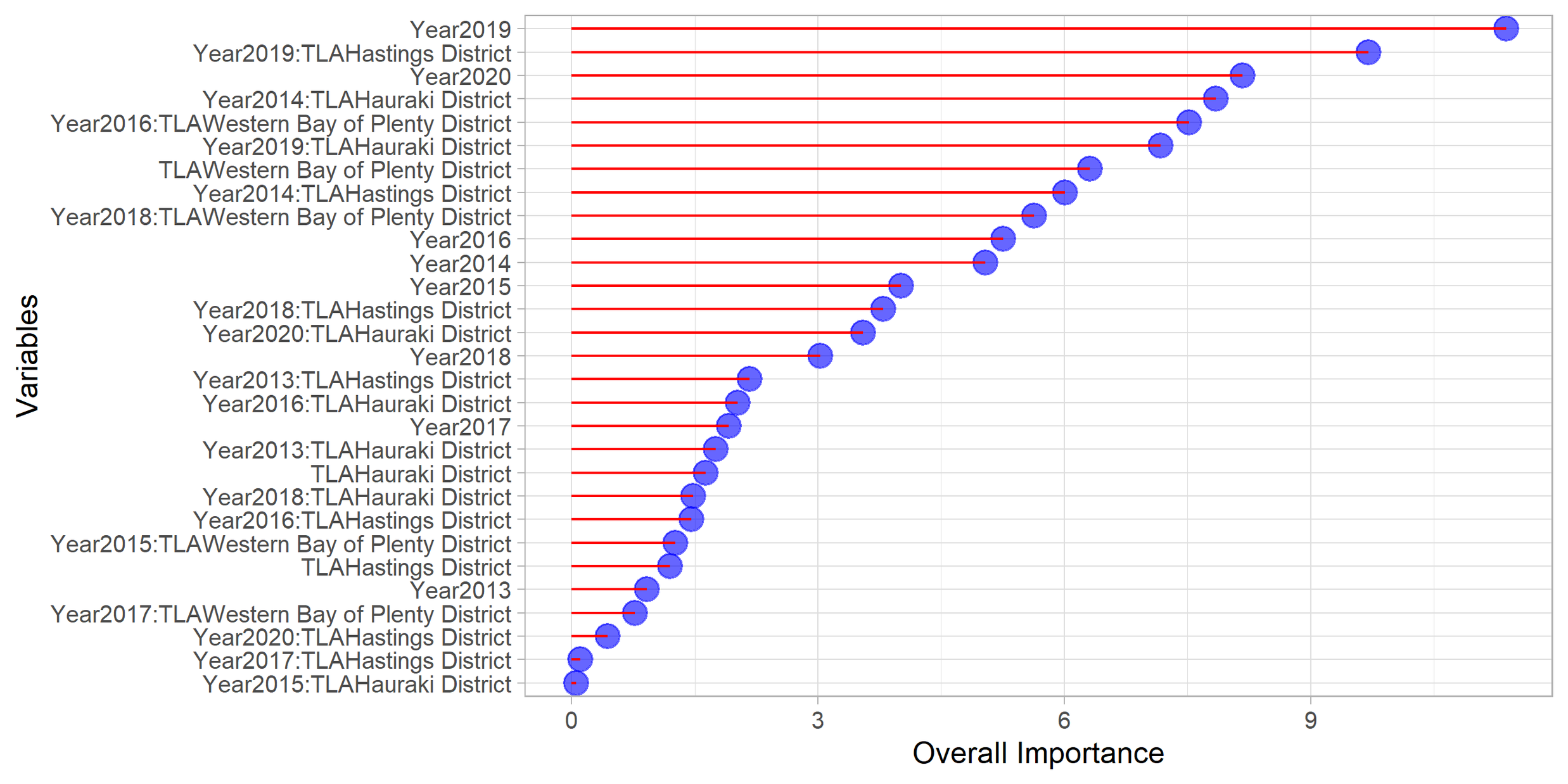

Supplement: S2 Fig — (TIF) [file pone.0303756.s002.tif]

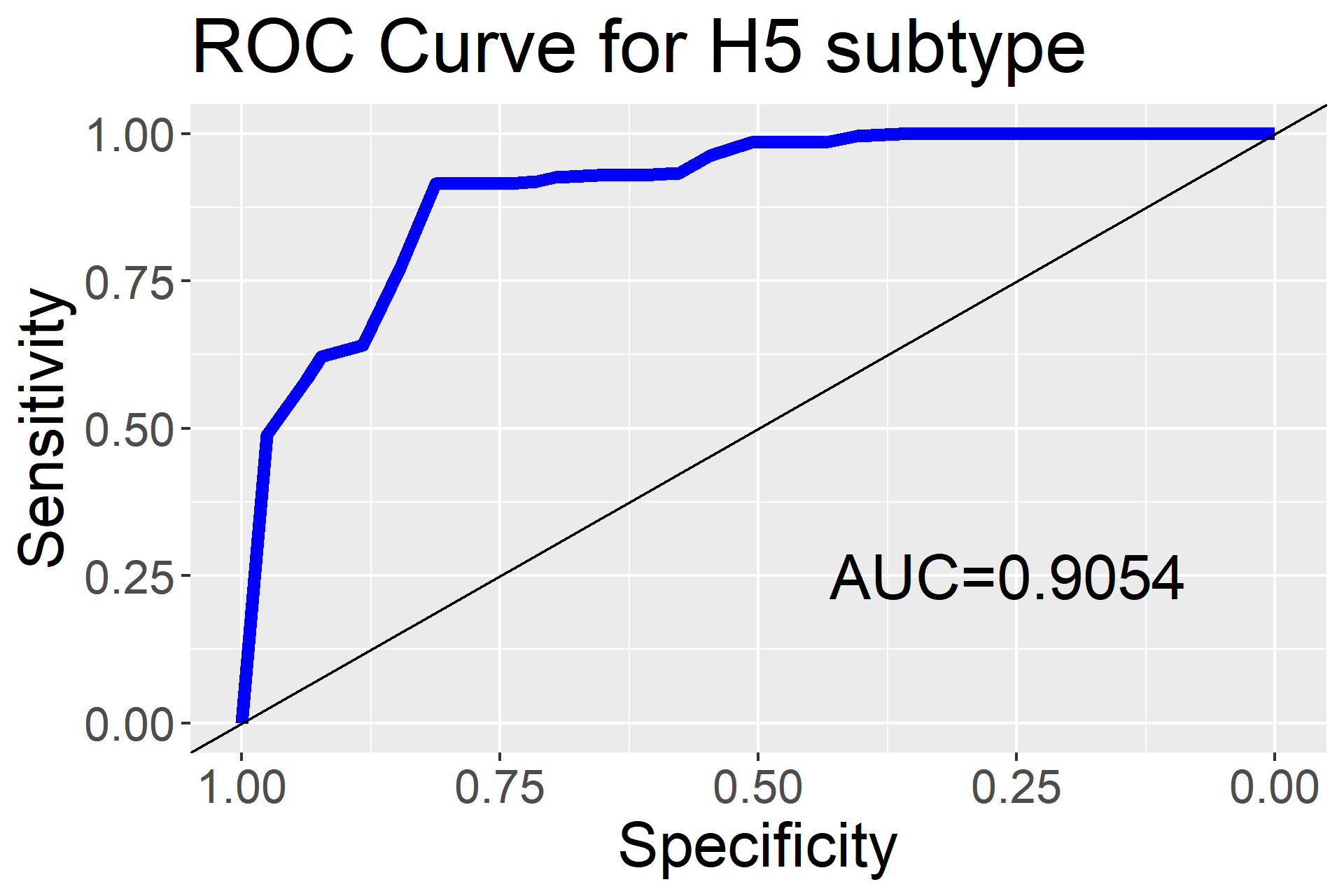

Supplement: S3 Fig — (TIF) [file pone.0303756.s003.tif]

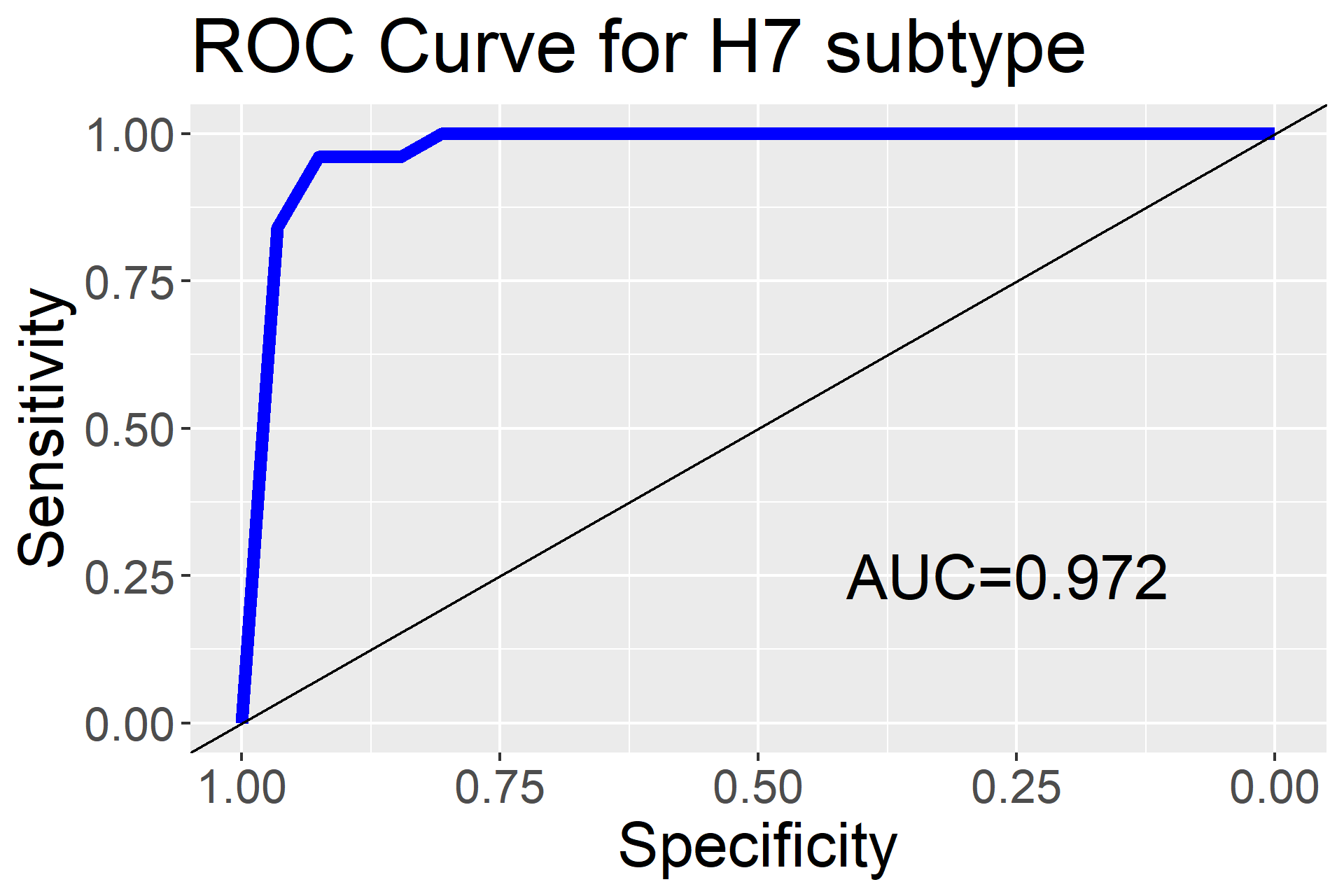

Supplement: S4 Fig — (TIF) [file pone.0303756.s004.tif]
