# Supplementary material for: Avian influenza viruses in New Zealand wild birds, with an emphasis on subtypes H5 and H7: Their distinctive epidemiology and genomic properties
Source: PLoS One. 2024 Jun 3;19(6):e0303756. doi: 10.1371/journal.pone.0303756 (PMC11146706; doi:10.1371/journal.pone.0303756)
Supplement: S1 Table — (DOCX) [file pone.0303756.s005.docx]

| Sample  collection year | Location of sample collection (District = Territorial Local Authority, TLA) | Date of sample collection | Species sampled | No. of samples collected | No. of samples/poll tested | No. of blood samples  collected | AIV isolates |
| --- | --- | --- | --- | --- | --- | --- | --- |
| Year 2004 |  |  |  |  |  |  |  |
|  | Western Bay of Plenty District | 01–03.02.04 | Mallard | 159c | 53 | 85 | c2xH1N2  c3xH2N3  c1xH3N2  c2xH4N6  c1xH11N1 |
|  | Southland District | 11.03.04 | Mallard | 72c | 24 | 32 | c1xH2N7 |
|  | Timaru District | 08.03.04 | Mallard | 47c | 16 | 32 | 0 |
|  | Waitaki District | 09.03.04 | Mallard | 67c | 23 | 34 | c1xH4N6 |
|  | Southland District | 10.03.04 | Mallard | 36c | 12 | 32 | c1xH4N6 |
|  | Hauraki District | 02.10.04 | Lesser knot  Godwit | 222c  66c | 74  22 |  | 0  0 |
| Year 2005 |  |  |  |  |  |  |  |
|  | Hasting District | 26.01.05 | Mallard | 117c | 39 | 30 | c1xH4N6  c1xH10N4 |
|  | Whakatane District | 01.02.05 | Mallard | 120c | 40 | 30 | 0 |
|  | Western Bay of Plenty District | 02.02.05 | Mallard | 123c | 41 | 30 | c1xH1N1  c1xH4N6 |
|  | Timaru District | 09–10.02.05 | Paradise and  mallard ducks | 49c  10c | 17  3 | 33 | 0  0 |
|  | Timaru District | 22.02.05 | Paradise and mallard ducks | 4c  156c | 1  53 | 0  34 | 0  c1xH6N2 |
|  | Southland District | 24.02.05 | Mallard | 79c | 26 | 33 | 0 |
|  | Southland District | 24.02.05 | Mallard | 66c | 22 | 33 | 0 |
|  | Invercargill City | 25.02.05 | Mallard | 82c | 28 | 34 | c1xH4N6  c1xH1N? |
|  | Hauraki District | 07.05.05 | Mallard | 170c* | 57 | 0 | c1xH7N7 c1xH6N9 |
|  | Hauraki District | 11.05.05 | Mallard | 134c* | 45 | 0 | c1xH4N6  c1xH11N9 |
|  | Hauraki District | 14.01.05 | Lesser knot  Godwit | 219c  65c | 73  22 | 0  0 | 0  0 |
| Year 2006 |  |  |  |  |  |  |  |
|  | Western Bay of Plenty District | 01–02.02.06 | Mallard | 160c | 54 | 39 | c2xH4N6  c1xH3N8 |
|  | Whakatane District | 01–02.02.06 | Mallard | 168c | 56 | 30 | c1xH3N8  c2xH4N6 |
|  | Hauraki District | 18.02.06 | Mallard | 240 c | 80 | 39 | c1xH4N2  c1xH10N4 |
|  | Hauraki District | 30.11.06 | Lesser knot  Godwit | 214  24 | 74  8 |  | 0  0 |
|  | Kaipara District | 14.12.06 | Lesser knot  Turnstone | 6  14 | 2  5 |  | 0  0 |
| Year 2007 |  |  |  |  |  |  |  |
|  | Hauraki District | 12–13.01.07 | Mallard | 209c | 70 | 34 | c11xH3N8  c1xH3N6 c1xH11N3 |
|  | Hauraki District | 12–13.01.07 | Mallard | 91c | 30 | 22 | c3xH1N2  c1xH3N8  c3xH4N6  c1xH4N7  c1xH5N7 |
|  | Hasting District | 22–23.01.07 | Mallard  Paradise ducks  Grey teal | 140c&72o  43c & 23o  50c & 31o | 47&24  15&8  17&11 | 41 | c4xH3N8  c4xH4N6  0  0 |
|  | Whakatane District | 07.03.07 | Mallard | 167 o&c | 56 | 48 | c1xH5N2  c1xH12N5 |
|  | Western Bay of Plenty District | 07–08.02.07 | Mallard | 218 o&c | 73 | 45 | c1xH2N9 |
|  | Invercargill City | 06.03.07 | Mallard | 200c | 67 | 57 | 0 |
|  | Hauraki District | 15–16.12.07 | Lesser knot  Godwit | 19o&c  29o&c | Not pooled | 0  0 | 0  0 |
| Year 2008 |  |  |  |  |  |  |  |
|  | Hauraki District | 11.01.08 | Mallard | 243c | 81 |  | c1xH3N6  c3xH4N6 |
|  | Hasting District | 22.01.08 | Mallard and  Paradise ducks | 122c  50c | 41  16 |  | c1xH3N8  c1xH4N8  c3xH4N6 |
|  | Rotorua District | 24.01.08 | Mallard | 121c | 40 |  | 0 |
|  | Whakatane District | 24.01.08 | Mallard | 122c | 41 |  | c3xH4N6 |
|  | Western Bay of Plenty District | 18.01.08 | Mallard | 196c | 65 |  | c2xH1N1  c2xH3N1  c3xH5N1  c2xH5N3 |
|  | Invercargill City | 07.02.08 | Mallard | 243c | 81 |  | c2xH3N8  c2xH5N1  c1xH5N2  c2xH11N2 |
|  | Hauraki District | 13. 01.08 | Lesser knot  Godwit | 226o&c  17o&c | 75  6 |  | 0  0 |
| Year 2009 |  |  |  |  |  |  |  |
|  | Hauraki District | 23.01.09 | Mallard | 210c | 70 |  | c2xH3N8  c1xH4N6  c1xH4N?  c1xH11N?  c1xH11N2 |
|  | Rotorua District | 28.01.09 | Mallard | 76c | 25 |  | c1xH3N8 |
|  | Western Bay of Plenty District | 27–28.01.09 | Mallard | 176o&c | 59x2 |  | c2xH3N8  c1xH3N2  c1xH6N6 |
|  | Hasting District | 29.01.09 | Mallard | 138o&c | 46x2 |  | c2xH4N? |
|  | Invercargill City | 02.02.09 | Mallard | 243o&c | 81x2 |  | c1xH1N1 |
|  | Timaru District | 02.05.09 | Mallard | 192o&c | 64x2 |  | 0 |
|  | Marlborough District | 22.12.09 | Black-billed gull  Black-backed gull | 200o&c  30o&c | 67x2 |  | 0  0 |
|  | Hauraki District | 21.11.09 | Lesser knot  Godwit | 69o&c  79o&c | 23x2  27x2 |  | 0  0 |
| Year 2010 |  |  |  |  |  |  |  |
|  | Gore District | 07–08. 01.10 | Black-billed gull | 210o&c | 70x2 |  | 0 |
|  | Hauraki District | 09.01.10 | Mallard | 167o&c | 56x2 |  | c3xH4N6  c1xH4N? |
|  | Whakatane District | 28.01.10 | Mallard | 98o&c | 33x2 |  | c1xH3N2 |
|  | Rotorua District | 28.01.10 | Mallard | 89o&c | 30X2 |  | 0 |
|  | Western Bay of Plenty District | 27.01.10 | Mallard | 231o&c | 77x2 |  | c1xH4N6  c1xH10N5 |
|  | Hasting District | 01.02.10 | Mallard | 210o&c | 70x2 |  | c2xH7H7 |
|  | Wellington District | 11.05.10 | Little blue penguin | 109o&c | 36x2 |  | 0 |
|  | Southland District | 04–08.02.10 | Yellow-eyed penguin | 54o&c | Not pooled |  | 0 |
|  | Kapiti district | 05.04.10 | Sooty shearwater | 80o&c | Not pooled |  | 0 |
|  | Marlborough District | 04.12.10 | Black- billed gull | 200o&c | 67x2 |  | 0 |
|  | Marlborough District | 11.12.10 | Black- backed gull | 202o&c | 67x2 |  | 0 |
|  | Southland District | 21.12.10 | Black-billed gull | 225o&c | 75x2 |  | 0 |
|  | Marlborough District | 21.11.10 | Black-backed gull | 86o&c | 29X2 |  | 0 |
|  | Hauraki District | 23–24.10.10 | Wrybill | 80o&c | 30X2 |  | 0 |
| Year 2011 |  |  |  |  |  |  |  |
|  | Hauraki District | 15.01.11 | Mallard | 240o&c | 80x2 |  | c1xH1N1 |
|  | Hasting District | 26.01.11 | Mallard | 231o&c | 77x2 |  | c1xH4N6 |
|  | Western Bay of Plenty District | 02–03.02.11 | Mallard | 320o&c | 107x2 |  | c1xH3N8  c2xH3N2  c1xH4N6  c2xH5N2  c1xH5N8  c1xH5N9  c1xH6N1  c1xH9N8 |
|  | Auckland District | 14.92.11 | Penguin | 12c | Not pooled |  | 0 |
| 2012 |  |  |  |  |  |  |  |
|  | Hauraki District | 14–15.01.12 | Mallard | 206o&c | Not Pooled |  | c2xH3N2  c1xH3N8  c2xH4N6  c2xH4N2  c1xH4N3  c1xH6N2  c1xH10N3  c1xH11N3 |
|  | Hasting District | 23.01.12 | Mallard | 238o&c | Not pooled |  | c2xH4N6 |
|  | Western Bay of Plenty District | 25.01.12 | Mallard | 320 o&c | Not pooled |  | c2xH3N2  c3xH4N2  c2xH4N6 |
| Year 2013 |  |  |  |  |  |  |  |
|  | Hauraki District | 08.01.13 | Mallard | 320o&c | Not pooled |  | c3xH4N6  c1xH10N7 |
|  | Rotorua District | 27–28.01.13 | Mallard | 100o&c | Not pooled |  | c3xH4N6 |
|  | Hasting District | 29–30.01.13 | Mallard | 240o&c | Not pooled |  | c3xH10N7 |
|  | Western Bay of Plenty District | 27–28.01.13 | Mallard | 300 o&c | Not pooled |  | c1xH3N6  c1xH4N2  c3xH4N6  c1xH4N7  c1xH4N9  c1xH5N2 |
| Year 2014 |  |  |  |  |  |  |  |
|  | Hauraki District | 03.01.14 | Mallard | 320 o&c | Not pooled |  | c2xH3N8  c4xH4N6 c1xH4N9 c1xH10N7 |
|  | Hasting District | 13.01.14 | Mallard | 240 o&c | Not pooled |  | c1xH3N8  c4xH4N6 |
|  | Western Bay of Plenty District | 23.01.14 | Mallard | 320 o& | Not pooled |  | c2x H3N8  c5x H4N6  o1x H10N7  c1x H10N7 c1x H11N9 |
| Year 2015 |  |  |  |  |  |  |  |
|  | Hauraki District | 06.01.15 | Mallard | 320 o&c | Not pooled |  | c1x H4N6  c1x H3N8  c1x H6N8 |
|  | Western Bay of Plenty District | 20.01.15 | Mallard | 320 o&c | Not pooled |  | c1x H1N1  c2x H4N6  c2x H6N2 |
|  | Gisborne District | 23–24.01.15 | Mallard | 220 o&c | Not pooled |  | c4x H4N6  c1x H3N8 |
|  | Waikato District | 13.06.15 | Mallard | 43 o&c | Not pooled |  | 0 |
|  | Wairoa District | 23–24.01.15 | Mallard | 100 o&c | Noy pooled |  | 0 |
|  | Southland District | 03–05.07.15 | Mallard | 40 o&c | Not pooled |  | o2x H7N7  c1x H7N7 |
|  | Southland District | 05–06.07.15 | Mallard | 22 o&c | Not pooled |  | o1x H7N7  c1x H7N7 |
| Year 2016 |  |  |  |  |  |  |  |
|  | Hauraki District | 05.01.16 | Mallard | 320 o&c | Not pooled |  | o1x H3N6  c1x H3N3  c1x H3N6  c1x H3N8  o1x H4N6  c8x H4N6  c2x H5N3  o5x H10N3  c8x H10N3  c1x H10N5  c1x H10N6  c1x H10N1 |
|  | Hasting District | 20.01.16 | Mallard | 129 o&c | Not pooled |  | o1x H4N6  c4x H4N6 |
|  | Gisborne District | 21.01.16 | Mallard | 194 o&c | Not pooled |  | c4x H4N6  c1x H6N2  c1x H11N3 c1x H11N9 |
|  | Western Bay of Plenty District | 22–23.01.16 | Mallard | 320 o&c | Not pooled |  | c2x H5N3 c1x H4N3  c1x H10N3 |
| 2017 |  |  |  |  |  |  |  |
|  | Auckland District | 21–22.01.17 | Mallard | 320 o&c | Not pooled |  | c2x H3N?  c5x H3N8  o2x H3N8  c1x H3N2  c1x H11N3  c2x H11N? |
|  | Gisborne District | 21.01.17 | Mallard | 300 o&c | Not pooled |  | o1xH4N2  c1xH4N2  c1xH4N8 |
|  | Western Bay of Plenty District | 25–26.01.17 | Mallard | 320 o&c | Not pooled |  | o1xH3N?  c4x H3N8  c1xH6N2  c1x H7N7 |
|  | Hasting District | 23.01.17 | Mallard | 100 o&c | Not pooled |  | c1xH6N2  o1xH4N6 |
|  | Invercargill City District | 14.02.17 | Mallard | 135 o&c | Not pooled |  | c1x H4N6  c2x H6N2 |
| 2018 |  |  |  |  |  |  |  |
|  | Hauraki District | 20.01.18 | Mallard | 300 o&c | Not pooled |  | c7x H3N1  c1x H6N6  c1x H7N5 |
|  | Hasting District | 18.01.18 | Mallard | 240 o&c | Not pooled |  | c1xH4N?  o1x H4N6 |
|  | Western Bay of Plenty District | 26–27.01.18 | Mallard | 320 o&c | Not pooled |  | o3x H3N2  c5x H4N6  cH11N2  cH11N? |
|  | Gisborne District | 24–25.01.18 | Mallard | 90 o&c | Not pooled |  | 0 |
| 2019 |  |  |  |  |  |  |  |
|  | Hauraki District | 12–13.01.19 | Mallard | 320 o&c | Not pooled |  | c1x H3N6 c1x H4N8  c1x H4N?  c1x H4N6  c1x H6N8  c1x H11N9 |
|  | Gisborne District | 24–25.01.19 | Mallard | 240 o&c | Not pooled |  | 0 |
|  | Hasting District | 24–25.01.19 | Mallard | 143 o&c | Not pooled |  | oH3N8 |
|  | Western Bay of Plenty District | 17–18.01.19 | Mallard | 320 o&c | Not pooled |  | c2x H3N2  c1x H3N?  c2x H3N?  c1x H4N6  c2x H4N? |
| 2020 |  |  |  |  |  |  |  |
|  | Hauraki District | 13.01.20 | Mallard | 320 o&c | Not pooled |  | c1x H4N6  c1x H5N2 o1x H11N9  c1x H1N? |
|  | Western Bay of Plenty District | 16–17.01.20 | Mallard | 320 o&c | Not pooled |  | c1 x H3N2  c2 x H3N8  c1x H3N8 |
|  | Hasting District | 27–28.01.20 | Mallard | 320 o&c | Not pooled |  | 0 |
|  | Waipa District | 01.02.20 | Mallard | 301 o&c | Not pooled |  | c1xH3N8  c1x H1N? |
