# Supplementary material for: Avian influenza viruses in New Zealand wild birds, with an emphasis on subtypes H5 and H7: Their distinctive epidemiology and genomic properties
Source: PLoS One. 2024 Jun 3;19(6):e0303756. doi: 10.1371/journal.pone.0303756 (PMC11146706; doi:10.1371/journal.pone.0303756)
Supplement: S2 Table — (DOCX) [file pone.0303756.s006.docx]

| **Host** | **Country** | **Maximum percentage identity** | **No. of matches** |
| --- | --- | --- | --- |
| Avian | New Zealand | 100.00 | 10 |
| Avian | USA | 89.13 | 73 |
| Avian | Canada | 88.98 | 10 |
| Avian | Japan | 87.64 | 1 |
| Avian | Guatemala | 87.00 | 1 |
