# Supplementary material for: Avian influenza viruses in New Zealand wild birds, with an emphasis on subtypes H5 and H7: Their distinctive epidemiology and genomic properties
Source: PLoS One. 2024 Jun 3;19(6):e0303756. doi: 10.1371/journal.pone.0303756 (PMC11146706; doi:10.1371/journal.pone.0303756)
Supplement: S3 Table — (DOCX) [file pone.0303756.s007.docx]

| **Host** | **Country** | **Maximum percentage identity** | **No. of matches** |
| --- | --- | --- | --- |
| Avian | New Zealand | 100.00 | 6 |
| Avian | UK | 86.84 | 13 |
| Avian | Germany | 86.44 | 12 |
| Avian | Israel | 86.39 | 1 |
| Avian | Egypt | 86.08 | 1 |
| Avian | China | 86.07 | 8 |
| Avian | USA | 86.03 | 4 |
| Avian | Sweden | 85.90 | 20 |
| Avian | Pakistan | 85.90 | 3 |
| Avian | Italy | 85.87 | 2 |
| Avian | South Africa | 85.85 | 2 |
| Avian | Hong Kong | 85.84 | 4 |
| Avian | Ireland | 85.78 | 2 |
| Avian | Zimbabwe | 85.61 | 3 |
| Avian | Netherlands | 85.49 | 4 |
| Avian | Mongolia | 85.44 | 1 |
| Avian | Japan | 85.37 | 2 |
| Avian | Spain | 85.32 | 1 |
| Avian | Korea | 85.31 | 1 |
| Avian | Taiwan | 85.28 | 1 |
