# Supplementary material for: Avian influenza viruses in New Zealand wild birds, with an emphasis on subtypes H5 and H7: Their distinctive epidemiology and genomic properties
Source: PLoS One. 2024 Jun 3;19(6):e0303756. doi: 10.1371/journal.pone.0303756 (PMC11146706; doi:10.1371/journal.pone.0303756)
Supplement: S4 Table — (DOCX) [file pone.0303756.s008.docx]

| Model | AIC |
| --- | --- |
| Null | 11201 |
| Year | 10348 |
| TLA | 10664 |
| Year + Territorial Authority | 9745 |
| Year + Territorial Authority + Year*Territorial Authority | 8861 |
