# Supplementary material for: Avian influenza viruses in New Zealand wild birds, with an emphasis on subtypes H5 and H7: Their distinctive epidemiology and genomic properties
Source: PLoS One. 2024 Jun 3;19(6):e0303756. doi: 10.1371/journal.pone.0303756 (PMC11146706; doi:10.1371/journal.pone.0303756)
Supplement: S5 Table — (DOCX) [file pone.0303756.s009.docx]

| Variable | Category | Estimate | SE | OR (95% CI) | p value | **p value (LRT**) |
| --- | --- | --- | --- | --- | --- | --- |
| Year | 2012 (Reference) |  |  |  |  |  |
|  | 2013 | -0.21 | 0.10 | 0.8 (0.66-0.98) | 0.03 | **<0.0001** |
|  | 2014 | -0.36 | 0.10 | 0.70 (0.58-0.85) | 0.00 |  |
|  | 2015 | -1.05 | 0.10 | 0.35 (0.29-0.43) | 0.00 |  |
|  | 2016 | 0.93 | 0.11 | 2.55 (2.07-3.13) | 0.00 |  |
|  | 2017 | 0.79 | 0.11 | 2.19 (1.76-2.73) | 0.00 |  |
|  | 2018 | -0.18 | 0.09 | 0.83 (0.69-1.01) | 0.06 |  |
|  | 2019 | -0.98 | 0.10 | 0.38 (0.31-0.48) | 0.00 |  |
|  | 2020 | -1.01 | 0.10 | 0.36 (0.30-0.44) | 0.00 |  |
