# Supplementary material for: Avian influenza viruses in New Zealand wild birds, with an emphasis on subtypes H5 and H7: Their distinctive epidemiology and genomic properties
Source: PLoS One. 2024 Jun 3;19(6):e0303756. doi: 10.1371/journal.pone.0303756 (PMC11146706; doi:10.1371/journal.pone.0303756)
Supplement: S6 Table — (DOCX) [file pone.0303756.s010.docx]

| Variable | Category | Estimate | SE | OR (95% CI) | p value | **p value (LRT**) |
| --- | --- | --- | --- | --- | --- | --- |
| Territorial  Authority | Gisborne District (Reference) |  |  |  |  |  |
|  | Hauraki District | 0.35 | 0.07 | 1.42 (1.24-1.64) | 0.00 | **<0.0001** |
|  | Hastings District | -0.28 | 0.08 | 0.75 (0.64-0.88) | 0.00 |  |
|  | Western Bay of Plenty District | 1.09 | 0.07 | 2.96 (2.57-3.41) | 0.00 |  |
