# Supplementary material for: Avian influenza viruses in New Zealand wild birds, with an emphasis on subtypes H5 and H7: Their distinctive epidemiology and genomic properties
Source: PLoS One. 2024 Jun 3;19(6):e0303756. doi: 10.1371/journal.pone.0303756 (PMC11146706; doi:10.1371/journal.pone.0303756)
Supplement: S7 Table — (DOCX) [file pone.0303756.s011.docx]

| Variables | Category | Estimate | SE | OR (95% CI) | p value | **p value (LRT)** |
| --- | --- | --- | --- | --- | --- | --- |
| Year | 2012 (Reference) |  |  |  |  |  |
|  | 2013 | -0.17 | 0.19 | 0.84 (0.58-1.22) | 0.37 | **<0.0001** |
|  | 2014 | 1.29 | 0.26 | 3.62 (2.22-6.08) | 0.00 |  |
|  | 2015 | -0.96 | 0.35 | 0.25 (0.13-0.50) | 0.00 |  |
|  | 2016 | -0.75 | 0.35 | 6.44 (3.27-13.16) | 0.00 |  |
|  | 2017 | 0.37 | 0.33 | 1.87 (1.00-3.61) | 0.06 |  |
|  | 2018 | -0.95 | 0.38 | 3.14 (1.52-6.71) | 0.00 |  |
|  | 2019 | -2.06 | 0.18 | 0.13 (0.09-0.18) | 0.00 |  |
|  | 2020 | -1.44 | 0.18 | 0.24 (0.17-0.33) | 0.00 |  |
| Territorial  Authority | Gisborne District (Reference) |  |  |  |  |  |
|  | Hauraki District | 0.55 | 0.34 | 1.73 (0.91-3.40) | 0.10 | **<0.0001** |
|  | Hastings District | -0.40 | 0.34 | 0.67 (0.35-1.31) | 0.23 |  |
|  | Western Bay of Plenty District | 1.72 | 0.27 | 5.61 (3.36-9.85) | 0.00 |  |
| Year*Territorial Authority | 2013-Hauraki District | -0.46 | 0.27 | 0.63 (0.38-1.06) | 0.08 |  |
|  | 2014-Hauraki District | -2.50 | 0.32 | 0.08 (0.04-0.15) | 0.00 | **<0.0001** |
|  | 2015-Hauraki District | -0.02 | 0.40 | 0.99 (0.44-2.11) | 0.96 |  |
|  | 2016-Hauraki District | 0.92 | 0.46 | 2.51 (1.03-6.14) | 0.04 |  |
|  | 2018-Hauraki District | -0.62 | 0.25 | 0.54 (0.23-1.21) | 0.00 |  |
|  | 2019-Hauraki District | 1.83 | 0.25 | 6.21 (3.78-10.26) | 0.00 |  |
|  | 2020-Hauraki District | 0.89 | 0.27 | 2.44 (1.49-4.01) | 0.03 |  |
|  | 2013-Hastings District | 0.59 | 0.34 | 1.80 (1.06-3.07) | 0.00 |  |
|  | 2014-Hastings District | -2.04 | 0.42 | 0.13 (0.07-0.25) | 0.14 |  |
|  | 2016-Hastings District | -0.62 | 0.43 | 0.54 (0.23-1.22) | 0.00 |  |
|  | 2018-Hastings District | -1.65 | 0.29 | 0.19 (0.08-0.44) | 0.66 |  |
|  | 2019-Hastings District | 2.76 | 0.34 | 15.84 (9.09-27.77) | 0.21 |  |
|  | 2020-Hastings District | 0.13 | 0.35 | 1.14 (0.64-2.00) | 0.00 |  |
|  | 2015-Western Bay of Plenty District | 0.43 | 0.30 | 1.53 (0.78-2.94) | 0.14 |  |
|  | 2016-Western Bay of Plenty District | -2.62 | 0.19 | 0.07 (0.04-0.14) | 0.36 |  |
|  | 2017-Western Bay of Plenty District | -0.26 | 0.26 | 0.77(0.40-1.47) | 0.00 |  |
|  | 2018- Western Bay of Plenty District | -2.09 | 0.35 | 0.12 (0.06-0.25) | 0.00 |  |

| Goodness of fit |  |
| --- | --- |
| Pseudo R^2^ (McFadden) | 0.2142 |
| AUC | 0.7943 |
