# Supplementary material for: Avian influenza viruses in New Zealand wild birds, with an emphasis on subtypes H5 and H7: Their distinctive epidemiology and genomic properties
Source: PLoS One. 2024 Jun 3;19(6):e0303756. doi: 10.1371/journal.pone.0303756 (PMC11146706; doi:10.1371/journal.pone.0303756)
Supplement: S8 Table — (DOCX) [file pone.0303756.s012.docx]

| Variables | Category | Estimate | SE | OR (95% CI) | p value | **p value (LRT)** |
| --- | --- | --- | --- | --- | --- | --- |
| Year | 2012 (Reference) |  |  |  |  |  |
|  | 2013 | -0.3265 | 5174.0441 | 0.7214 (0.0000-Inf) | 1.0000 | **<0.0001** |
|  | 2014 | -0.3053 | 5144.8104 | 0.7369 (0.0000-Inf) | 1.0000 |  |
|  | 2015 | -0.1796 | 4999.2812 | 0.8356 (0.0000-Inf) | 1.0000 |  |
|  | 2016 | -0.1836 | 5003.6523 | 0.8323 (0.0000-Inf) | 1.0000 |  |
|  | 2017 | 21.2842 | 3738.0165 | 1752362826.5277 (0.0000-Inf) | 0.9955 |  |
|  | 2018 | 20.1418 | 3738.0163 | 559090252.1183 (0.0000-Inf) | 0.9957 |  |
|  | 2019 | -0.1079 | 4922.4633 | 0.8977 (0.0000-Inf) | 1.0000 |  |
|  | 2020 | 17.0356 | 3738.0165 | 25030690.0574 (0.0000-Inf) | 0.9964 |  |
| Territorial Authority | Gisborne Area (Reference) |  |  |  |  |  |
|  | Hauraki District | 21.1038 | 2796.2403 | 1462994057.2368 (0.0000-Inf) | 0.9940 | **<0.0001** |
|  | Hastings District | 0.9687 | 3633.8569 | 2.6344 (0.0000-Inf) | 0.9998 |  |
|  | Western Bay of Plenty District | 17.9975 | 2796.2401 | 65498818.2387 (0.0000-Inf) | 0.9949 |  |

| Goodness of fit |  |
| --- | --- |
| Pseudo R^2^ (McFadden) | 0.3818 |
| AUC | 0.9720 |
